# The microRNA-211-5p/P2RX7/ERK/GPX4 axis regulates epilepsy-associated neuronal ferroptosis and oxidative stress

**DOI:** 10.1186/s12974-023-03009-z

**Published:** 2024-01-08

**Authors:** Xueying Li, Pusheng Quan, Yao Si, Fei Liu, Yuwei Fan, Feifan Ding, Lina Sun, Han Liu, Shuo Huang, Linlin Sun, Fan Yang, Lifen Yao

**Affiliations:** 1 Department of Neurology, The First Affiliated Hospital, Harbin Medical University, Harbin, 150081 China; 2 Department of Neurology, The Affiliated Hospital of Inner Mongolia Medical University, Hohhot, Inner Mongolia China

**Keywords:** Epilepsy, microRNA-211-5p, P2RX7, Ferroptosis, Bioinformatics technology, MAPK signaling pathways

## Abstract

Ferroptosis is an iron-dependent cell death mechanism involving the accumulation of lipid peroxides. As a critical regulator, glutathione peroxidase 4 (GPX4) has been demonstrated to be downregulated in epilepsy. However, the mechanism of ferroptosis in epilepsy remains unclear. In this study, bioinformatics analysis, analysis of epilepsy patient blood samples and cell and mouse experiments revealed strong associations among epilepsy, ferroptosis, microRNA-211-5p and purinergic receptor P2X 7 (P2RX7). P2RX7 is a nonselective ligand-gated homotrimeric cation channel, and its activation mainly increases neuronal activity during epileptic seizures. In our study, the upregulation of P2RX7 in epilepsy was attributed to the downregulation of microRNA (miR)-211-5p. Furthermore, P2RX7 has been found to regulate GPX4/HO-1 by alleviating lipid peroxidation induced by suppression of the MAPK/ERK signaling pathway in murine models. The dynamic decrease in miR-211-5p expression induces hypersynchronization and both nonconvulsive and convulsive seizures, and forebrain miR-211-5p suppression exacerbates long-lasting pentylenetetrazole-induced seizures. Additionally, in this study, induction of miR-211-5p expression or genetic-silencing of P2RX7 significantly reduced the seizure score and duration in murine models through the abovementioned pathways. These results suggest that the miR-211-5p/P2RX7 axis is a novel target for suppressing both ferroptosis and epilepsy.

**Supplementary Information:**

The online version contains supplementary material available at 10.1186/s12974-023-03009-z.

## Highlights

1. We first utilized bioinformatics technology to screen microRNA-211-5p reduction in epileptic patients and verified this in epileptic patient blood samples and mice hippocampal tissue.
2. We first uncovered a significant interaction exists between microRNA-211-5p and P2RX7.
3. We first verify that microRNA-211-5p exerts an influence on epilepsy by regulating ferroptosis through targeting P2RX7.
4. Our data provide comprehensive evidence that microRNA-211-5p/P2RX7/ERK/GPX4 pathway could be a promising target for the treatment of epilepsy.

**Supplementary Information:**

The online version contains supplementary material available at 10.1186/s12974-023-03009-z.

## Introduction

Epilepsy is a complex neurological disorder with a heterogeneous aetiology affecting 50 million individuals annually worldwide. Seizures can be triggered by various factors that could perturb the physiological structures or functions of the brain [1, 2]. Unfortunately, approximately 30% of patients with epilepsy are resistant to pharmacological treatment [3], and a significant proportion (30%) of them are classified as ‘of unknown cause’ [4]. Even worse, no therapeutic drugs can effectively prevent the onset or progression of epilepsy. Therefore, there is an urgent need to explore new therapeutic targets and develop novel drugs that can delay or prevent the onset, inhibit the progression and mitigate the associated comorbidities of epilepsy [5].

MicroRNAs (miRs) are small noncoding RNAs that can regulate mRNA expression levels by interacting with the 3′-untranslated regions (3′UTRs) of their target genes [6]. Physiologically, miRs are involved in regulating various biological processes, such as neuronal development [7], endoplasmic reticulum (ER) stress [8] and mitochondrial function [9]. Thus, miRs may serve as potential therapeutic targets for human diseases. Specifically, miR-211-5p regulates neuronal differentiation and viability in the brain and inhibits neurite growth and branching in vitro [10]. Moreover, dynamic decreases in miR-211-5p expression induce hypersynchronization, leading to both nonconvulsive and convulsive seizures. Suppression of forebrain miR-211-5p further exacerbates long-lasting pentylenetetrazole-induced seizures [11], underscoring the potential role of miR-211-5p in the development of epilepsy. Interestingly, our bioinformatics analysis revealed a decrease in miR-211-5p expression in patients with epilepsy. However, the precise mechanism by which miR-211-5p influences the occurrence and progression of epilepsy remains incompletely understood.

The degradation of haemoglobin in the brain leads to the deposition of haemosiderin, which is closely linked to neurological disorders including epilepsy [12]. Iron overload has been considered a primary cause of refractory epilepsy resulting from haemorrhagic stroke. Chronic seizures following the injection of haemoglobin or iron salts (ferric chloride, FeCl3) into the cortex have been observed in rats. These free radicals may lead to lipid peroxidation in neuronal membranes, ultimately inducing epilepsy [13]. These findings suggest that iron is critically involved in the pathogenesis of epilepsy. In addition, another form of programmed cell death, known as ferroptosis, is characterized by the iron-dependent accumulation of free radicals and lipid oxidation products [14]. Unlike apoptosis and autophagy, morphological changes in ferroptosis are highlighted by an increased density of the mitochondrial membrane [15]. Excess cytoplasmic iron can trigger lipid peroxidation, facilitating the formation of toxic lipid free radicals, as well as the initiation of ferroptosis [16]. Of all organs, the brain is most susceptible to oxidative stress [17], which can participate in epileptic seizure-induced neuronal cell death [18–20]. In fact, reducing oxidative stress with a variety of compounds (such as antioxidants and NADPH oxidase inhibitors) might prevent seizure-induced neuronal death [21–23]. Deletion of GPX4, an essential regulator of ferroptosis, has been found to induce susceptibility to epilepsy [24]. Furthermore, the downregulation of GPX4 in epileptic mice and patient blood sample was validated in our research. Nevertheless, systematic research on the mechanism of ferroptosis in epilepsy remains lacking, while research has been performed for other nervous system diseases. Therefore, it is necessary to explore how ferroptosis and oxidative stress occur during the development and progression of epilepsy.

P2RX7 is a nonselective ligand-gated homotrimeric cation channel activated by extracellular adenosine triphosphate (ATP), which has been proposed as a possible drug target [25]. For example, P2RX7 activation mainly occurs under pathological conditions involving high ATP release [26, 27], such as inflammation or increased neuronal activity (e.g., during epileptic seizures) [28]. In addition, P2RX7 receptor signaling components may serve as biomarkers for the diagnosis of temporal lobe epilepsy (TLE) [29]. In our study, we showed that the expression of P2RX7 was significantly increased in epilepsy. Moreover, we found that the upregulation of P2RX7 in epilepsy was attributed to the downregulation of microRNA-211-5p in the preliminary bioinformatics analysis and experiments of this study. It is still unclear whether there is a regulatory relationship between P2RX7 and ferroptosis, and whether they affect the progression of epilepsy.

In this research, we identified multiple mechanisms of ferroptosis in the pathogenesis of epilepsy and clarified that miR-211-5p accounts for the increase in P2RX7. Furthermore, we confirmed that P2RX7 is a novel regulator that inhibits ferroptosis by regulating the MAPK/ERK signaling pathway during the development and progression of epilepsy. These results provide clues for new therapeutic targets and options for epilepsy patients.

## Materials and methods

### Patient selection and blood collection

All blood (plasma) samples from healthy controls (N = 30) and epileptic patients (N = 60) were collected at the First Affiliated Hospital of Harbin Medical University (Heilongjiang, China) with the approval of the Institutional Review Board (IRB) of the Ethics Committee. All patients had a detailed clinical assessment, and were refractory to anti-seizure medication (multidrug therapy) prior to admission. Peripheral venous blood samples were collected from each subject who was in a fasting state into a Vacutainer tube containing potassium EDTA. Then, peripheral blood mononuclear cells (PBMCs) were immediately isolated using Ficoll density gradient centrifugation. The clinical data of the epilepsy patients are presented in Additional file 1: Table S1.

### Animals

Male C57BL/6 mice (aged 6–8 weeks, weighing at 20–30 g) were purchased from Harbin Medical University. All mice were housed in an indoor environment with a 12 h light/12 h dark cycle with free access to food and water. Experimental protocols for animals were approved by the Institutional Animal Care and Use Committee (IACUC) of Harbin Medical University.

### Virus construction and preparation

The recombinant adeno-associated virus (AAV) vectors for transgenes P2RX7 and green fluorescent protein (GFP) were manufactured by Shanghai GeneChem Co., Ltd. (Shanghai, China). A universal scrambled sequence containing mismatched bases served as a negative control. The sequences of the control shRNA and P2RX7-shRNA were 5′-CGCTGAGTACTTCGAAATGTC-3′ and 5′-GCATCTTTGACACTGCAGACT-3′, respectively. The shRNA target sequences were inserted into GV478 lentivectors, which were transfected into 293T cells. Afterwards, viral supernatants were harvested after 48 h, with a final virus titre of 5.81E+12 v.g./ml.

### Intrahippocampal injections and grouping

The mice were anaesthetized with 1% pentobarbital sodium (40 mg/kg, i.p.), fixed on a stereotactic instrument and then injected with AAV-P2RX7 (Sh-P2RX7 group), AAV-blank (Sh-Con group), PD98059 (ERK inhibitor), SB203580 (P38 inhibitor) or SP600125 (JNK inhibitor), respectively, into the hippocampus. Briefly, injections were performed using a stereotaxic frame to guide a micropipette into the hippocampus (bregma, 2.2 mm; lateral, 2.2 mm; ventral, 1.8 mm) according to the standard stereotaxic atlas. The micropipette was held in place for an additional 10 min before being slowly withdrawn. The incision was closed with sutures. Body temperature was maintained at 37 °C using a heating pad. To assess the efficiency of AAV-mediated knockdown, four mice were randomly chosen from each group and sacrificed on the 28th day after AAV injection. The hippocampus was immediately isolated and prepared for either scanning detection under confocal microscopy or Western blot analysis.

The remaining mice were stereotactically injected with kainic acid (KA, 25 mg/kg) to construct murine models of epilepsy. KA was injected slowly for 10 min and positioned in the hippocampus (AP-2.2 mm, ML-2.2 mm, V-1.8 mm). After injection, the needle was left in place for 10 min to avoid drug reflux. Two hours later, the mice received diazepam (10 mg/kg) to terminate seizures. The mice were randomly divided into eleven groups: (1) Control (n = 10, sham operation, received 1 μl PBS injection); (2) Epilepsy (n = 8, received KA injection); (3) Sh-P2RX7 + KA (n = 9, received successive injections of AAV-P2RX7 and KA); (4) Sh-Con + KA (n = 8, received successive injections of AAV-blank vectors and KA); (5) sh-P2RX7 + PD98059 + KA (n = 6, received successive injections of AAV-P2RX7, PD98059 [an ERK inhibitor] and KA); (6) sh-P2RX7 + SB203580 + KA (n = 7, received successive injections of AAV-P2RX7, SB203580 [a p38 inhibitor] and KA); (7) sh-P2RX7 + SP600125 + KA (n = 6, received successive injections of AAV-P2RX7, SP600125 [a JNK inhibitor] and KA); (8) miR-211-5p agomir + KA (n = 6, received successive injections of miR-211-5p agomir [a miR-211-5p activator, 5 nmol/g/day] and KA); (9) NC agomir + KA (n = 6, received successive injections of NC agomir [a agomir blank vectors, 5 nmol/g/day] and KA); (10) miR-211-5p antagomir + KA (n = 5, received successive injections of miR-211-5p antagomir [a antagomir inhibitor, 10 nmol/g/day] and KA) and (11) NC antagomir + KA (n = 5, received successive injections of NC antagomir [a antagomir blank vectors, 10 nmol/g/day] and KA). PD98059 (HY-12028), SB203580 (HY-10256) and SP600125 (HY-12041) were purchased from MedChemExpress (MCE). The miR-211-5p agomir, NC agomir, miR-211-5p antagomir and NC antagomir were designed by RiboBio, China. We collected the hippocampal tissue from mice 28 days after KA injection for our experiments.

### Electroencephalography (EEG) measurement

After 14 days of KA injection, two bipolar tungsten electrodes (Cat No. 796000, A-M Systems) and a bipolar stainless-steel electrode were respectively implanted into the bilateral ANT and the CA1 region of the region of the right hippocampus of mouse. EEG data were collected and analyzed using NicoletOne EEG System (parameters: 1–70 Hz low- and high-frequency filter, 15 mm/s recording speed; Natus). According to the Racine scale, spontaneous recurrent seizures (SRSs) on a scale of 4–5 and their duration were observed and recorded.

### Behavioural observation

Behavioural changes in epileptic mice at 90 min after KA injection were continually observed. Seizure activity was scored using the criteria described by Racine [30]. Seizure stages were classified as follows: stage 0, no response; stage 1, ear and facial twitching; stage 2, myoclonic jerks (MJs); stage 3, clonic forelimb convulsions; stage 4, generalized clonic seizures with turning to a side position; and stage 5, generalized tonic‒clonic seizures (GTCSs) or death. Mice with at least three consecutive seizures of stage 4 or 5 were defined as fully kindled.

### Transmission electron microscopy (TEM)

Mice were perfused with 2% paraformaldehyde and 2% glutaraldehyde in 0.1 mol/l sodium cacodylate buffer. The hippocampal tissue was fixed in phosphate-buffered glutaraldehyde (2.5%) and osmium tetroxide (1%). The hippocampal samples were cut, stained en bloc with 2% uranyl acetate (UA), dehydrated in acetone solutions at increasing concentrations, and embedded in an epoxy resin. The sections (70–90 nm thick) were stained with lead citrate and uranyl acetate. Ultrastructural images were then captured with a transmission electron microscope JEM1200 (JEOL, Tokyo, Japan). Images of the hippocampal mitochondria were obtained using 20× and 40× objective lenses.

### Haematoxylin and eosin (H&E) and Nissl staining

Murine hippocampal tissue was fixed in 4% paraformaldehyde and embedded in paraffin. The paraffinized brains were cut into 5 μm-thick sections and the sections were dewaxed and rehydrated according to the standard protocols.

1. For H&E staining, the protocol was as follows: (1) mouse tissue sections were immersed into a Coplin jar containing hematoxylin and agitate for 30 s; (2) the slices were rinsed in H_2_O for 1 min; (3) the slices were stained with a 1% eosin solution for 10–30 s with agitation;
2. For Nissl staining, mouse tissue sections were immersed in staining solution for 6 min and slightly differentiated with 1% glacial acetic acid at room temperature. The rection was terminated by washing with tap water. The degree of differentiation was assessed under a microscope.

For transparent mounting, the slices were dehydrated with ethanol, washed with distilled water, clarified with xylene, sealed with neutral gum, and examined under a microscope (Olympus Corporation, Tokyo, Japan). Images of the hippocampus were captured using a 20× objective lens.

### Perls’ iron staining

Paraffin slices (10 μm-thick) were cut from murine brains and incubated with xylene I and xylene II for 15 min, followed by hydration with 100%, 95%, 85%, 80%, 75% and 70% alcohol solutions for 1 min, each. Perls’ iron stain was prepared as a mixture of 80 ml 20% HCl and 80 ml 10% potassium ferrocyanide for 5 min. The tissue was incubated in the solution for 20–30 min and washed with distilled water 3 times. After eosin staining for 1 min, rapid dehydration was performed at 80%, 85%, 90% and 100% alcohol concentrations for 5 s, respectively. Finally, the slices were cleaned with xylene and sealed with resin. Images of the hippocampus were captured using a 10× objective lens.

### Cell lines and culture conditions

Immortalized murine hippocampal HT22 cells [31] (Bena Culture Collection, China) and human neuroblastoma SH-SY5Y cells, which have been widely used for studying oxidative stress [32] and epilepsy model [33] (ATCC, Manassas, VA, USA) were cultured in DMEM (C11995500BT, Gibco, USA) containing 10% FBS, 100 units of penicillin and 100 μg/ml streptomycin, and maintained at 37 °C in a 5% CO_2_ incubator.

### Cell transfection

Transfection was performed to upregulate or downregulate the mRNA expression levels of miR-211-5p and P2RX7. Briefly, miR-211-5p mimic, mimic negative control, miR-211-5p inhibitor, inhibitor negative control, siRNA-P2RX7, and siRNA-negative control were designed by RiboBio, China. According to the manufacturer’s protocol, each solution was added to Lipofectamine 3000 transfection reagent. The mixtures were equilibrated for 5 min at room temperature. Then, SH-SY5Y cells were transfected with the transfection mixture in serum-free cell medium for 48 h. The concentration of miR-211-5p mimic, mimic negative control and siRNA-P2RX7, or siRNA-negative control was 50 nM. The concentration of miR-211-5p inhibitor or inhibitor negative control was 100 nM.

### Cell death assay

SH-SY5Y cells were cultured in a 6-well plate and exposed to erastin (S7242, Selleck). Erastin is a ferroptosis inducer through reactive oxygen species (ROS) and iron-dependent signaling [34, 35]. The cells were divided into four groups: Control, Erastin (10 μM, 24 h), si-P2RX7 + Erastin, and si-Control + Erastin. After incubation with erastin for 24 h, PI and Hoechst 33342 were added at a concentration of 5 μg/ml for 10 min, each. Then, the cell death rate was measured by PI (+)/Hoechst (+). The percentage of cell death was determined as previously described [36].

### Dual-luciferase reporter assay

The plasmids containing the wild-type miR-211-5p-P2RX7 (wtLuc-P2RX7) response element and its corresponding mutant (mut-Luc-P2RX7) were purchased from Shanghai Genechem Co., Ltd. Plasmid DNA (wt-Luc-P2RX7, mut-Luc-P2RX7) and miR-211-5p mimic or miR-211-5p negative controls were co-transfected into 293T cells. Then, luciferase activity was assessed with a Double-Luciferase Reporter Assay Kit (Shanghai Genechem Co., Ltd) using the Dual-Light Chemiluminescent Reporter Gene Assay System, and normalized to firefly luciferase activity.

### GEO dataset collection

Public miRNA, mRNA, and single-cell sequencing datasets were downloaded from the GEO (Gene Expression Omnibus) website (http://www.ncbi.nlm.nih.gov/geo/) (GSE99455, GSE133554, GSE88992, GSE134697 and GSE140393). The GSE99455 [37, 38] dataset included hippocampal miRNA profiling of 16 patients with medically intractable epilepsy and 8 postmortem healthy controls. Among 36 samples in the GSE134697 dataset [39], 17 hippocampal and 17 neocortex samples were obtained from patients with drug-resistant temporal lobe epilepsy, whereas 2 neocortex samples were obtained from healthy subjects. Single brain cells from temporal tissues of 5 patients with temporal lobe epilepsy (TLE) were analyzed in GSE140393 [40, 41]. Single nuclei of the CA1 area from 2 control and 2 epileptic mice were downloaded from the GSE143560 dataset [42]. Six hippocampal samples from epileptic mice and 5 samples from control mice, each with three replicates were included in the GSE133554 dataset (https://www.ncbi.nlm.nih.gov/geo/query/acc.cgi?acc=GSE133554). The GSE88992 dataset [43] comprised 9 control hippocampus samples and 8 TLE samples from murine models.

### Single-cell raw data quality control

We analyzed single-cell RNA sequencing data from the GEO database containing temporal tissues of epileptic patients (accession: GSE 143560, GSE140393) using R (version 4.1.3) or the Seurat R package (version 4.1.1). For the initial QC step, cells with expression of < 200 or > 6000 genes, mitochondrial transcript proportion > 10%, and < 200 or > 20,000 unique molecular identifiers (UMI) were filtered out of the analysis. The R package Double Finder was used to identify potential doublets [44]. The samples were normalized using the Seurat SCTransform and combined using Seurat CCA integration workflow.

### Cell type identification

The FindClusters function of the Seurat package was used to identify clusters. The FindAllMarkers function (adjusted P < 0.05 and |logFC| > 0.25) was used to determine specific genetic markers associated with each cluster. Then, T-distributed Random Neighbour Embedding (tSNE) plots were used to visualize different cells. Specifically, excitatory neurons were marked by GRIN2A, inhibitory neurons by KCNIP1, microglia by CD74, astrocytes by AQP4, oligodendrocyte precursor cells (OPCs) by VCAN, oligodendrocytes (ODCs) by MOBP, pericytes by PDGFRB, and endothelial cells by CLDN5.

### Ferroptosis scores

A ferroptosis-related gene set was obtained from the FerrDb database (http://www.zhounan.org/ferrdb/). The ferroptosis activity of an individual cell was estimated with the R package AUCell (version 1.12.0). The AUCell is a statistical method to determine if a given gene set is enriched at the top quantile of a ranking gene signature for a single cell. As such, cells expressing more genes in the gene-set have higher AUC values. The “AUCell explore Thresholds” function was utilized to select an appropriate threshold for a given gene set, and to score respective enrichment in each cell. A score greater than the threshold value was defined as a “High Ferroptosis Score”. Subsequently, cell clustering tSNE plots were generated and colour-coded according to the AUC scores, so that the distribution of ferroptosis scores might be displayed in different cellular subsets.

### Differential gene expression analysis and functional enrichment

To assess differential gene expression (DEG), we executed the FindAllMarkers function integrated into the Seurat framework, employing the Wilcoxon test. Subsequently, Bonferroni correction was applied to adjust the associated p-values for multiple testing. The DEGs were then subjected to filtering, requiring a minimum log-fold change of 0.25 and a maximum adjusted p-value of 0.05. These refined DEGs were subsequently ranked based on their average log-fold change and false discovery rate (FDR).

Functional enrichment analysis of these DEGs was carried out utilizing the clusterProfiler package (v4.8.0). The gene sets used for this enrichment analysis were curated from Gene Ontology terms and Kyoto Encyclopedia of Genes and Genomes (KEGG) pathways. Enrichment analysis was performed separately for both KEGG and Gene Ontology terms using the enrich KEGG and enrich GO functions. In Fig. 6a, we conducted enrichment analysis using differentially expressed transcription factors and differentially expressed surface proteins derived from the DEGs. This choice was made because transcription factors and surface proteins serve as essential mediators for regulating gene expression and cellular interactions with the external environment.

### Western blotting (WB)

The mice were administered 1% pentobarbital sodium and decapitated. Then, bilateral hippocampal tissues were immediately collected. SH-SY5Y cells were collected after exposure. Cells and lysed tissues in RIPA buffer supplemented with protease and phosphatase inhibitors were collected. Protein concentrations were measured with BCA reagent (P0006, Beyotime Biotechnology, China). Protein samples (20 μg) were separated by SDS‒PAGE (10% separation gel) and transferred to a PVDF membrane (Merck and Co., Inc., Whitehouse Station, NJ, United States, Germany). After blocking with TBST containing 5% nonfat milk for 1 h, the membranes were incubated with Gpx4 (ab125066, 1:1000; Abcam), Hmox-1 (ab189491, 1:2000; Abcam), P2rx7 (ab259942, 1:1000, Abcam), phospho-Erk1/2 (AF1015, 1:1000; Affinity Biosciences), Erk1/2 (AF0155, 1:1000; Affinity Biosciences), phospho-p38 (AF4001, 1:1000; Affinity Biosciences), p38 (AF6456, 1:1000; Affinity Biosciences), phospho-Jnk (AF3318, 1:1000; Affinity Biosciences), and Jnk (AF6318, 1:1000; Affinity Biosciences) overnight at 4 °C. The next day, following three washes in TBST, the membranes were incubated with horseradish peroxidase (HRP)-conjugated anti-rabbit IgG (A0208, 1:2000, Beyotime) or anti-mouse IgG (A0216, 1:2000, Beyotime) at room temperature for 1 h. The protein levels were normalized to GAPDH (#5174, 1:1000, Cell Signaling Technology).

### Total RNA isolation and quantitative polymerase chain reaction

A stereotactic technique was applied to inject drugs into the murine hippocampus. All male C57BL/6 mice were randomly divided into three groups: (1) Control (n = 5, sham operation, received 1 μl PBS injection); (2) Epilepsy (n = 5, received KA injection); and (3) Fer-1 (n = 5, received Fer-1 injection). Two hours after KA injection, the mice received diazepam (10 mg/kg) to terminate seizures. Then, the hippocampus was immediately isolated, and total RNA was extracted using TRIzol reagent (Invitrogen, United States) following the manufacturer’s protocol. Total RNA was quantified using a spectrophotometer (Nanodrop 2000, Thermo Fisher, USA). All RNA samples presented 260/280 nm ratios between 1.8 and 2.0.

The RNA samples were shipped to Suzhou Transcriptome Biotechnology Co., Ltd. (Suzhou, China). The mRNA was enriched by oligo(dT) beads. After concentrations were determined with an adaptor-specific q-PCR kit, equimolar samples were pooled and clustered for sequencing with HiSeq2000 (Illumina, USA).

Differential gene expression analysis was carried out using the DESeq2 package [45]. Differences with a *p* value < 0.05 and log2|fold change| > 0.585 were considered significant.

### Real-time quantitative PCR

For miRNA quantification, Bulge-loop™ miRNA qRT‒PCR Primer Sets (one RT primer and a pair of qPCR primers for each set) specific for miR-211-5p and u6 were designed by RiboBio (Guangzhou, China). After drug treatment, total RNA from tissues or cell cultures was extracted using TRIzol reagent (Thermo Fisher Scientific) following the manufacturer’s protocols. Then, 1 μg of total RNA was reverse-transcribed using the Revert Aid First Strand cDNA synthesis kit (#K1622; Thermo Fisher Scientific). Real-time PCR was performed using the double-stranded DNA dye SYBR Green (#RR037A; Takara Biotechnology, Dalian, China) on an ABI7500 platform. All samples were analyzed in triplicate, and gene expression was normalized to GAPDH. The sequences of primers used to detect specific genes are listed in Additional file 1: Table S2.

### Measurement of dihydroethidium (DHE)

After drug treatment, DHE (10.0 μM, 1.5 ml) was added to each well for 30 min and incubated at 37 °C. The samples were ultimately observed under fluorescence microscopy (Olympus, Tokyo, Japan).

### Measurement of malondialdehyde (MDA), superoxide dismutase (SOD) and glutathione (GSH) levels

MDA, SOD and GSH levels were tested in SH-SY5Y cells and murine tissues using colorimetric assays (S0131, S0103, and S0053, Beyotime) according to the manufacturer’s protocol.

### Immunofluorescence (IF) staining

Mice were anaesthetized and perfused through the left cardiac ventricle. The brains were removed and postfixed overnight in 4% paraformaldehyde. Frozen sections were prepared using a cryostat microtome with a thickness of 7-µm for IF staining. Next, tissue sections and cells were permeabilized with 0.25% Triton X-100 in PBS for 10 min, and washed 3 times with PBS before blocking with 1% BSA for 60 min. After rinsing with PBS, tissue sections and cells were incubated with primary anti-P2RX7 (28207-1-AP, 1:400, Proteintech), anti-Iba1(ab283319, 1:100, Abcam), anti-GFAP (#3670, 1:50, Cell Signaling Technology) and anti-NeuN (66836-1-lg, 1:100, Proteintech) antibodies. Each sample was incubated with a secondary antibody for 1 h at RT. After staining the nucleus with DAPI, images were captured using fluorescence microscopy (Olympus, Japan). Images of the hippocampus were captured using a 40× objective lens.

### Statistical analysis

Statistical analysis was performed by GraphPad Prism 7 software (GraphPad, San Diego, CA, United States). Student’s t test was used to compare variables between two groups. One-or two-way analysis of variance (ANOVA) was used to analyze differences among groups followed by LSD post hoc comparisons when appropriate. All images are representative results from a minimum of three independent replicates with similar trends. All data are presented as the mean ± standard deviation (SD). A value of p < 0.05 (2-tailed) was considered statistically significant.

## Results

### MiR-211-5p is downregulated in the blood of epilepsy patients and in the hippocampal tissue of epileptic mice, and it targets P2RX7

**Fig. 1:**
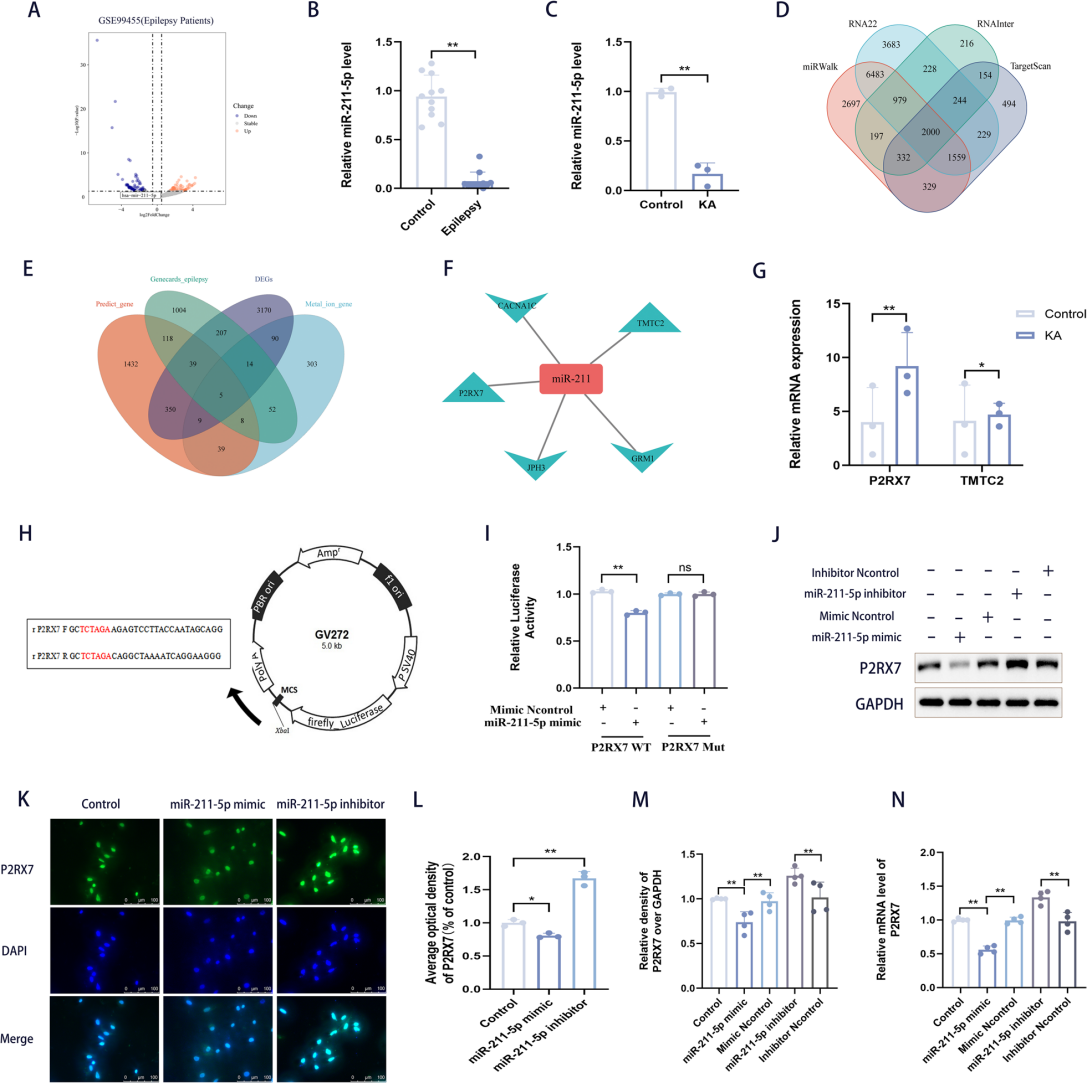
MiR-211-5p is downregulated in the blood of epilepsy patients and in the hippocampal tissue of epileptic mice, and it targets P2RX7. **A** The transcriptome data of GSE99455 abstracted from hippocampal miRNA profiling of intractable epilepsy and healthy controls. Differential expression analysis in epilepsy vs. control revealed 70 upregulated genes and 74 downregulated genes (miR-211-5p was significantly downregulated, LogFC = − 1.384, p = 0.0013). **B** miR-211-5p was downregulated in venous blood samples of 60 epileptic patients. **C** miR-211-5p was downregulated in the hippocampus of KA-induced epileptic mice. **D** The Venn diagram overlapped 14,576 DEGs in the miRWalk database, 15,405 DEGs in the RNA22 database, 4350 DEGs in the RNAInter database and 5341 DEGs in the TargetScan database, and 2000 potential miRNA-211-5p target genes were predicted. To further narrow down the number of target genes. **E** The Venn diagram overlapping 2000 predicted miR-targeted genes, 1447 DEGs in epilepsy related genes in the Genecards database, 3884 DEGs in the GSE134697 database and 520 DEGs in the Metal Ion Gene database, and highlights the five most promising genes regulated by miRNA-211-5p, including (**F**) upregulated P2RX7 and TMTC2, and downregulated CACNA1C, JPH3 and GRM1. **G** The relative mRNA levels of P2RX7 and TMTC2 in KA-induced epileptic mice compared with control mice were measured by qRT-PCR. **H** A construction diagram of target double-luciferase reporter genes (with mutant sequences labelled in red). **I** Relative luciferase activities were detected in cells after co-transfection with empty vector, P2RX7-WT or P2RX7-Mut, and miR-211-5p mimics or NC. **J**, **M** SH-SY5Y cells were transfected with mir-211-5p mimic (50 nM) vs. inhibitor (100 nM) for 48 h. Western blots and quantification of changes in relative protein levels of P2RX7 in mimic-miR-211-5p vs. inhibit-miR-211-5p SH-SY5Y cells. P2RX7 was decreased in the mimic-miR-211-5p group but increased in the inhibit-miR-211-5p group. **K**–**L** Images and quantification of immunofluorescence staining revealed the level of P2RX7 in mimic-miR-211-5p vs. inhibit-miR-211-5p SH-SY5Y cells. (Scale bar, 100 μM). **N** The relative mRNA expression of P2RX7 in mimic-miR-211-5p vs. inhibit-miR-211-5p SH-SY5Y cells compared with control cells. All data are expressed as the mean ± SD. *p < 0.05, **p < 0.01, ns, no significance

For the preliminary screening of related mechanisms and target genes in epilepsy, GSE99455 (including hippocampal miRNA profiles of 16 patients with intractable epilepsy and 8 postmortem healthy controls) from the GEO database was selected for analysis. To identify the target miRNAs, miRNA differential expression analysis was performed. A total of 144 differentially expressed miRNAs were screened. Notably, miR-211-5p (LogFC = − 1.384, p = 0.0013) was confirmed to be downregulated in epilepsy patients (Fig. 1A). Moreover, the GSE99455 dataset included information on 16 patients (including 5 childhood-onset, 6 adolescent-onset, and 5 adult-onset patients) with medically intractable epilepsy who were divided into adult-onset (Additional file 2: Fig. S1A) and adolescence-onset (Additional file 2: Fig. S1B) groups, and miR-211-5p was also confirmed to be downregulated in adult-onset and adolescence-onset epilepsy patients vs. controls (Additional file 2: Fig. S1A, B). Furthermore, downregulation of miRNA-211-5p was validated in blood samples from epileptic patients (the clinical data of epilepsy patients are presented in Additional file 1: Table S1) and in hippocampal tissues of mice with kainic acid (KA)-induced epilepsy (Fig. 1B, C). Subsequently, to identify potential effector genes of miRNA-211-5p involved in epilepsy, we further explored the intersection with differentially expressed genes (DEGs) in the miRWalk, RNA22, RNAInter, and TargetScan databases, and 2000 genes were selected (Fig. 1D). To narrow down the list of candidate genes for experimental validation, the above 2000 genes, genes from DEGs (3884 genes), epilepsy-related genes in Genecards (1447 genes), and genes in “GOBP METAL ION HOMEOSTASIS. V2022.1. Hs” (520 genes) from MsigDB (http://software.broadinstitute.org/gsea/msigdb/) were analyzed. Ultimately, 5 target genes were obtained (Fig. 1F), of which P2RX7 and TMTC2 were upregulated and CACNA1C, JPH3 and GRM1 were downregulated in epilepsy. MiRs bind to complementary mRNAs and prevent their translation, resulting in a decrease in their protein levels. Therefore, we focused only on these upregulated genes in epilepsy. qRT-PCR was performed to detect differential levels of P2RX7 and TMTC2 in epileptic mice. Compared with TMTC2, P2RX7 was significantly upregulated (Fig. 1G). These results suggest that P2RX7 may play a role in epilepsy.

Based on the interaction network illustrated, the RNA sequence alignment showed that the 3′-UTR of P2RX7 mRNA contained a complementary site for the seed region of miRNA-211-5p as shown in Supplement File (Additional file 2: Fig. S1C). The direct interaction between miR-211-5p and P2RX7 was verified by a double-luciferase gene reporting assay (Fig. 1H). In the P2RX7-Wt group, the luciferase activities were significantly repressed by miR-211-5p overexpression compared with the mimic control group. However, this effect was not observed in the mutated P2RX7 groups (Fig. 1I). Moreover, miR-211-5p was overexpressed or knocked down at the cellular level (Additional file 2: Fig. S1D, E). WB (Fig. 1J, M), IF (Fig. 1K, L) and qRT-PCR (Fig. 1N) experiments also confirmed these results. Thus, we hypothesized that the upregulation of P2RX7 in epilepsy was attributed to the downregulation of microRNA-211-5p.

### P2RX7 expression is upregulated in the hippocampal tissue and excitatory neurons of an epilepsy mouse model

**Fig. 2:**
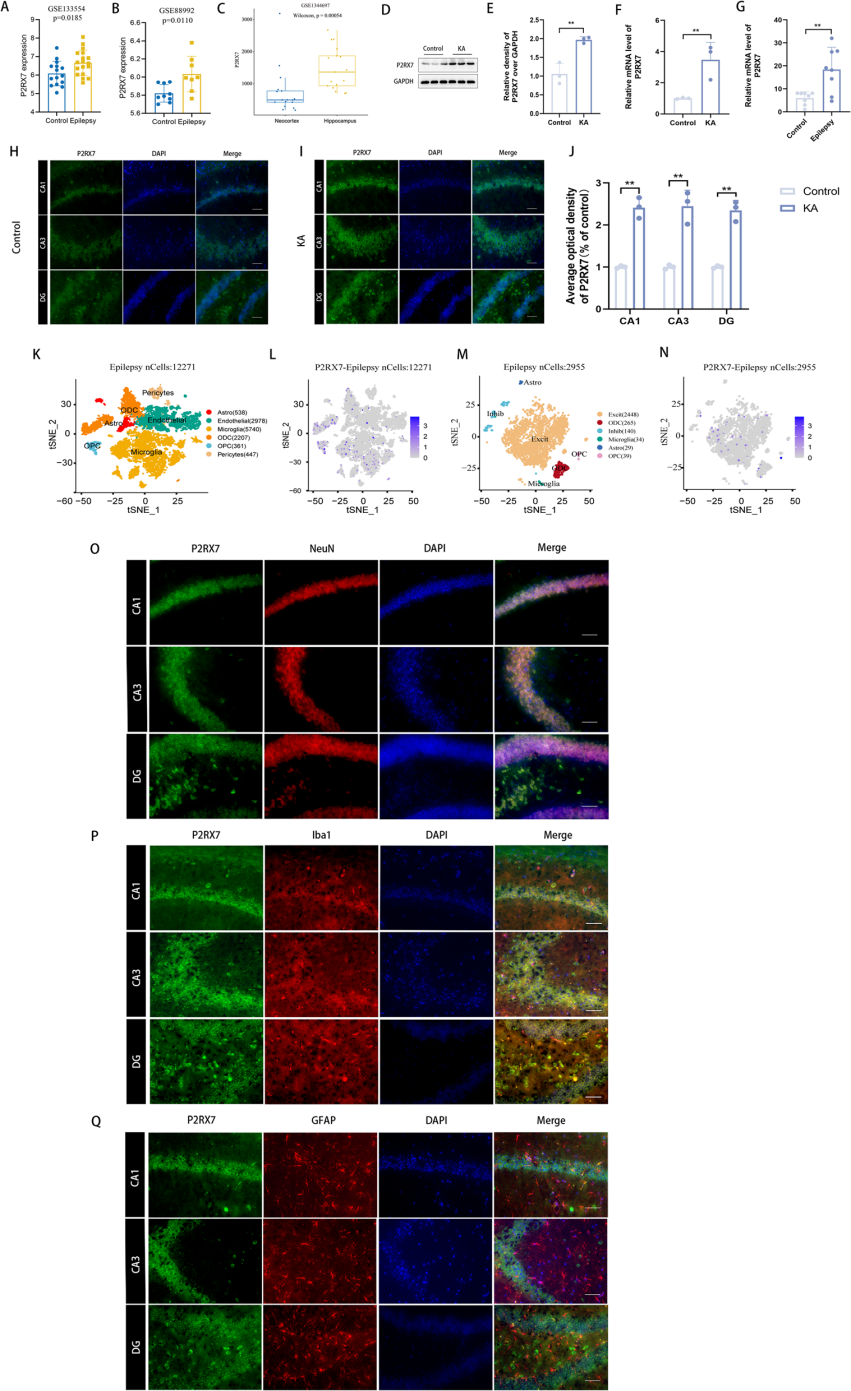
P2RX7 expression is upregulated in the hippocampal tissue and excitatory neurons of an epilepsy mouse model. **A**–**C** Elevated P2RX7 expression in epileptic mice compared with controls (**A**, **B**) as well as in the hippocampus compared to the neocortex in epilepsy (**C**). The GSE133554 dataset (**A**, p = 0.0185) included 6 hippocampal samples from epileptic mice and 5 samples from control mice, each with three replicates. The GSE88992 dataset (**B**, p = 0.0110) comprised 9 control hippocampus samples and 8 TLE samples derived from mice. The GSE1344697 dataset (**C**, p = 0.00054) included 17 hippocampal and 17 neocortex samples from patients with drug-resistant TLE, and 2 neocortex samples from healthy subjects. **D**, **E** Western blots and quantification of the protein levels of P2RX7 in KA-induced epileptic mice and control mice. P2RX7 was upregulated in the KA group compared to the control group. **E** The relative mRNA expression levels of P2RX7 in hippocampal tissues were evaluated by qRT-PCR in KA-induced epileptic mice, compared to control group mice. **F** Relative mRNA expression levels of P2RX7 in blood samples were evaluated by qRT-PCR in epilepsy patients compared to non-diseased controls. **G**–**J** Representative images from IF staining presenting P2RX7 expression in murine hippocampal sections in the KA group and control group. Data are presented as the mean ± SD of three independent experiments. Scale bars = 100 μm. **K** The GSE140393 database was performed on 12,271 single cells after quality control, and 25 cellular clusters were identified and divided into 6 major cell types in the epilepsy groups. **L** P2RX7 expression at the single-cell level in GSE140393. **M** The GSE143560 database was used to analyze 6422 single nuclei after quality control, and 17 cellular clusters were identified and divided into 6 major cell types in the epilepsy groups. P2RX7 was mainly expressed in excitatory neurons. **N** P2RX7 expression at the single-cell level in GSE143560. **O**–**Q** Representative images from multiple immunofluorescences staining highlight P2RX7 in neurons (NeuN, **O**), microglia (Iba-1, **P**) and astrocytes (GFAP, **Q**). Scale bars = 100 μm. All data are expressed as the mean ± SD. *p < 0.05, **p < 0.01

To investigate the role of P2RX7 in epilepsy, GSE133553 (including 6 hippocampus samples from epileptic mice and 5 samples from control mice) and GSE88992 (including 8 epileptic mouse hippocampus samples and 9 control mouse hippocampus samples) from the GEO database were selected for analysis. Among them, P2RX7 (Fig. 2A, p = 0.0185) (Fig. 2B, p = 0.0110) was confirmed to be upregulated. Seizures, whether isolated or recurrent, originate from the brain’s cerebral cortex or hippocampus region. The hippocampus plays a crucial role in the initiation and conduction of epileptic seizures and is referred to as the transfer station and amplifier of seizure activities [46]. Additionally, in GSE133554 (including 6 epileptic mouse samples and 5 control mouse samples), P2RX7 (Fig. 2C, p = 0.00054) was confirmed to be upregulated in the hippocampus compared to the neocortex. This profile was verified in human blood samples by qRT-PCR (Fig. 2G) and in hippocampal tissue from KA-induced epileptic mice by WB (Fig. 2D, E), qRT-PCR (Fig. 2F, G) and IF (Fig. 2H–J).

Under normal physiological conditions, P2RX7 expression in the brain seems to be mainly restricted to glial cells including microglia [47] and oligodendrocytes [48]. P2RX7 has also been suggested to be expressed on neurons [25] and astrocytes [49]. The cell type in which P2RX7 is increased following status epilepticus (SE) and during epilepsy is, however, still a matter of debate. For systematic research, single-cell sequencing analysis from GSE140393 (including single brain cells from temporal tissues of 5 epileptic patients) was performed on 12,271 single cells after quality control. Afterwards, 25 cellular clusters were identified and divided into 6 major cell types, including 5740 microglia, 538 astrocytes, 361 OPC, 2207 ODC, 447 pericytes and 2978 endothelial cells (Fig. 2K). This cell-set had no neurons due to sample preparation, and the samples were sourced solely from epileptic patient. Here, P2RX7 was mainly expressed in microglia (Fig. 2L). In addition, GSE143560 (including single nuclei of the CA1 area from 2 epileptic mice and 2 control mice) was performed on 6422 single nuclei after quality control. Interestingly, 17 cellular clusters were identified and divided into 6 major cell types, including 5490 excitatory neurons (Excit), 325 inhibitory neurons (Inhib), 57 microglia, 53 astrocytes, 63 oligodendrocyte precursor cells (OPCs), and 434 oligodendrocytes (ODCs) (Fig. 2M). P2RX7 was expressed predominantly in excitatory neurons (Fig. 2N) in epilepsy. In addition, multiple immunofluorescence staining showed that P2RX7 was primarily expressed in excitatory neurons, followed by microglia, and was rarely expressed in astrocytes (Fig. 2O–Q). These bioinformatic and experimental results indicate that P2RX7 may primarily play a role in excitatory neurons in an epilepsy model. As a supplement, WB was used to explore the expression of P2RX7 in the immortalized murine HT22 cell line and human SH-SY5Y cell line. The expression of P2RX7 was very low in HT22 cells (Additional file 2: Fig. S1F, G). Thus, SH-SY5Y cells were selected for subsequent experiments.

### P2RX7 knockdown exerts anticonvulsant effects and attenuates hippocampal neuronal loss in a mouse model

**Fig. 3:**
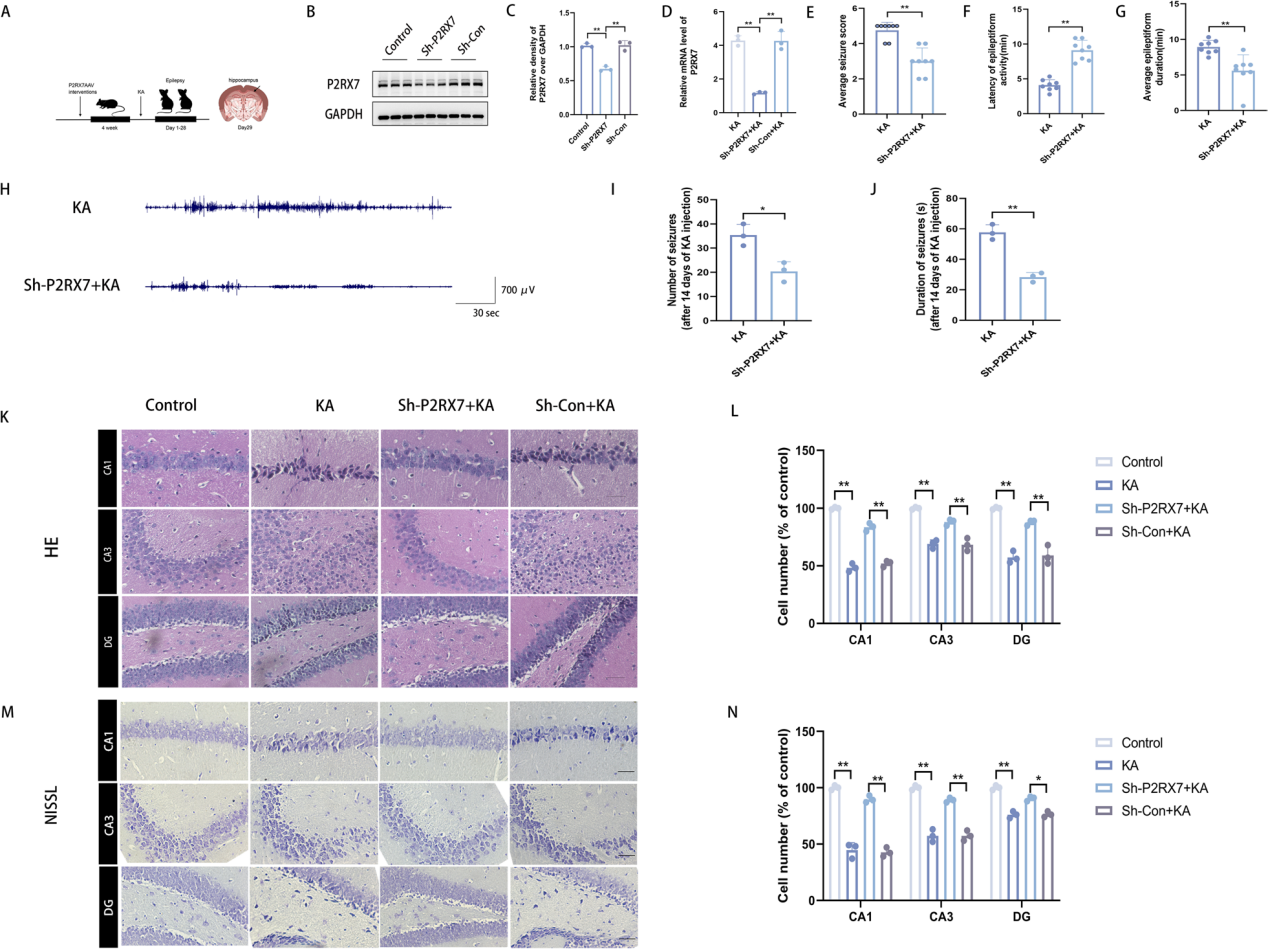
P2RX7 knockdown exerts anticonvulsant effects and attenuates hippocampal neuronal loss in a mouse model. **A** A schematic diagram of murine models. **B**, **C** Western blots and quantification for comparing the protein levels of P2RX7 in control mice, sh-P2RX7 mice and sh-Con mice. P2RX7 was downregulated in the sh-P2RX7 group compared to the control and sh-Con groups. **D** The relative mRNA levels of P2RX7 in the sh-P2RX7 group compared to the control and the sh-Con groups. **E**–**G** The average seizure-stage score reached 4.75 ± 0.46 in the KA group vs. 3.00 ± 0.76 in the sh-P2RX7 + KA group (**E**). **F** The latency of epileptiform activity (**F**, 9.13 ± 1.42 in the sh-P2RX7 + KA group vs. 4.09 ± 0.80 in the KA group) and an average epileptiform duration of the first seizure (**G**, 5.59 ± 2.24 in sh-P2RX7 + KA group vs. 8.92 ± 0.95 in KA group). **H** Representative 5-min EEG recordings from the hippocampus (after 14 days of KA injection) showed spontaneous electrographic seizures in the KA and sh-P2RX7 + KA group. **I** The number of SRS recorded after 14 days of KA injection, demonstrating that the sh-P2RX7 + KA group had a lesser number of seizures than those of the KA group. **J** The mean SRSs duration of sh-P2RX7 + KA group was significantly shorter than that of the KA group. **K**–**M** Representative images of H&E and Nissl staining in the CA1, CA3 and DG areas. **L**, **N** Semiquantitative analysis of cell number (% of control). Scale bars = 100 μm. All data are expressed as the mean ± SD. *p < 0.05, **p < 0.01

To demonstrate the impact of P2RX7-knockdown on the progression of epilepsy in vivo, a P2RX7 recombinant adeno-associated virus (P2RX7-AAV) targeting neuron-specific expression was injected into the hippocampus, and a KA-induced epilepsy model was constructed after 4 weeks (Fig. 3A). WB and qRT-PCR showed that the expression of P2RX7 was significantly decreased compared to that in the Control group, indicating successful hippocampal knockdown of P2RX7 protein (Fig. 3B–D). The consecutive seizure stage score, the latency of epileptiform seizures and the epileptiform duration of the first seizure are the main manifestations of epilepsy. To evaluate the anticonvulsant effect of P2RX7 knockdown, seizure severity scores were assessed by the Racine scale, and the latency to achieve full kindling and epileptiform duration of the first seizure were recorded. The mean seizure severity scores, latency and duration were not significantly different between the KA group and the sh-Con + KA group, indicating that the virus vector exerted no effect on the KA-induced epilepsy model. Compared with the KA group, the sh-P2RX7 + KA group showed a significant decrease in the average seizure score (3.00 ± 0.7 vs. 4.75 ± 0.46) (Fig. 3E) and in the average epileptiform duration of the first seizure (5.59 ± 2.24 vs. 8.92 ± 0.95) (Fig. 3G), whereas there was a significant increase in the latency of epileptiform activity (9.13 ± 1.42 vs. 4.09 ± 0.8) (Fig. 3F). Following hippocampal injection, three mice injected with AAV (one with sh-P2RX7 and two with sh-Con) died, presumably because of the excessive anaesthetic dose. Three additional mice were added as replacements to maintain the same total number of animals in the experimental groups. Following hippocampal injections of KA, 80% of the mice in the KA group without AAV injection and the sh-Con + KA group survived, while 90% of the mice in the sh-P2RX7 + KA group survived. We finally had 10 animals in the Control group, 9 in the sh-P2RX7 + KA group, and 8 in the KA and sh-Con + KA groups. Next, hippocampal tissues of from mice at 8 weeks were subjected to histochemical staining to elucidate the mechanism of P2RX7. In addition to observing seizures in mice within 120 min after KA injection, we also confirmed the results by performing electroencephalogram (EEG) testing on mice 14 days after KA injection (Fig. 3H). The mean number of SRSs in the sh-P2RX7 + KA group was reduced compared to the KA group (Fig. 3I). The mean seizure duration of the sh-P2RX7 + KA group was significantly shorter than that of the KA group (Fig. 3J). Based on H&E and Nissl staining, KA changed the morphology of the hippocampal CA1, CA3 and DG areas of brain tissue compared with that in the Control group: loss of pyramidal cells and Nissl bodies, pyknosis of nuclei, and disordered and irregular arrangement of pyramidal cell layers (Fig. 3K–N). The pathological changes and neuron loss in the hippocampal CA1, CA3 and DG areas of brain tissue in the sh-P2RX7 + KA group were significantly attenuated. These results show that P2RX7 knockdown exerts anticonvulsant effects and attenuates hippocampal neuronal loss in a mouse model.

### P2RX7 knockdown can reduce ferroptosis and oxidative stress in vitro

**Fig. 4:**
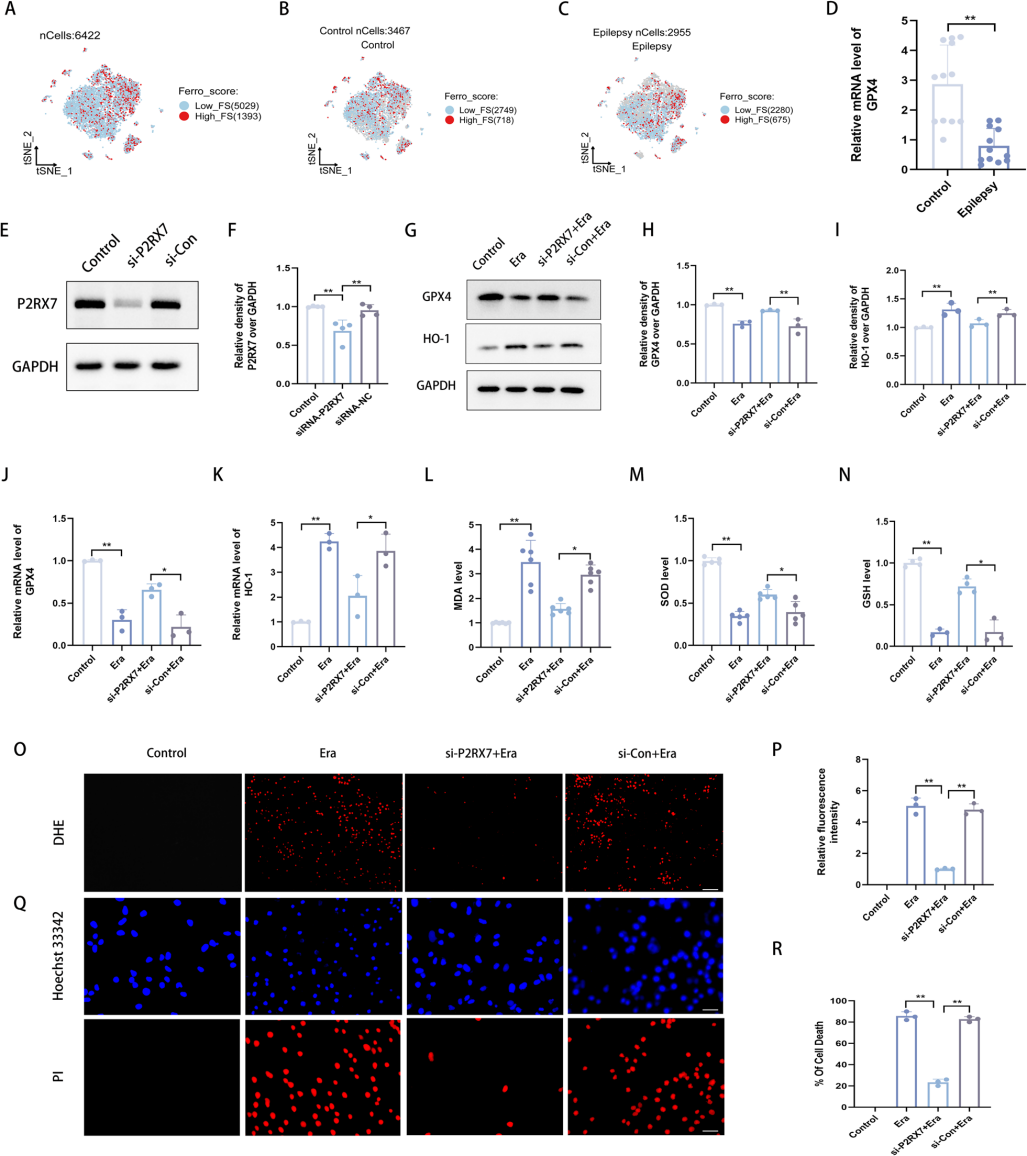
P2RX7 knockdown can reduce ferroptosis and oxidative stress in vitro. **A** Based on the GSE143560 database (6422 single nuclei after quality control), a tSNE plot of each cell based on the ferroptosis activity score as calculated by AUCell in the control and epilepsy groups. Cells with high-ferroptosis-scoring are highlighted in red. The 6422 single nuclei were divided into Low-Ferro score (Low-FS) (5029 cells) and High-Ferro score (High-FS) (1393 cells) expression groups. Meanwhile, the 6422 single nuclei were divided into control group (3467 cells) (**B**) and an epilepsy group (2955 cells) (**C**). Among 1393 cells, the proportion of cells with high ferroptosis activity in the epilepsy group (675/2955, 22.84%) (**C**) was greater than that in the control group (718/3467, 20.71%) (**B**). **D** Representative quantification of relative mRNA levels of GPX4 in blood samples from epilepsy patients and non-diseased controls. GPX4 was downregulated in epilepsy group compared to control group. **E**, **F** SH-SY5Y cells were transfected with P2RX7 (100 nM) for 48 h. Representative quantification of relative protein levels of P2RX7 in control, si-P2RX7 and si-Con groups. P2RX7 was decreased in the si-P2RX7 group compared to the control and si-Con groups. **G**–**I** Western blots and quantification of the protein levels of GPX4 and HO-1 in the control, Erastin, si-P2RX7 + Erastin and si-Con + Erastin groups. **J**, **K** Representative quantification of the relative protein and mRNA levels of GPX4 and HO-1 in the control, Erastin, si-P2RX7 + Erastin and si-Con + Erastin groups. **L**–**N** MDA, SOD and GSH assays to detect lipid peroxidation levels in the control, Erastin, si-P2RX7 + Erastin and si-Con + Erastin groups. **O**, **P** Representative images of SH-SY5Y cells by DHE (**O**) and semiquantitative analysis of relative fluorescence intensity (**P**). **Q**, **R** Representative images of SH-SY5Y cells by HO/PI staining (**Q**) and semiquantitative analysis of the percentage of cell death (**R**). All data are expressed as the mean ± SD. *p < 0.05, **p < 0.01

To investigate the anticonvulsant mechanism of P2RX7, GSE143560 (including 6422 single nuclei) was selected for further analysis. Although some evidence has confirmed the role of ferroptosis in the pathogenesis of epilepsy and that reversing the downregulation of GPX4 can effectively delay the course of epilepsy [50, 51], the existence of ferroptosis in epilepsy still needs more verification. Subsequently, to identify the potential mechanism, the single-cell sequencing data were divided into Low-Ferro score (Low-FS) (5029 cells) and High-Ferro score (High-FS) (1393 cells) groups (as shown in method 2.16) (Fig. 4A). Among 1393 cells, the proportion of high ferroptosis activity in the epilepsy group (675/2955, 22.84%) (Fig. 4C) was greater than that in the control group (718/3467, 20.71%) (Fig. 4B). In addition, a greater proportion of excitatory neurons in the epilepsy group presented high-ferroptosis activity (Fig. 4B, C). The core component of ferroptosis, GPX4, was decreased in blood samples of epileptic patients (Fig. 4D). As an in vitro cell line for ferroptosis activity in vitro, SH-SY5Y cells were chosen to validate the results of bioinformatics analysis. A ferroptosis model was successfully established by stably stimulating erastin (a ferroptosis inducer through reactive oxygen species and iron dependents signaling) [52]. To determine the relationship between P2RX7 and ferroptosis, siRNA of P2RX7 was constructed in SH-SY5Y cells (Fig. 4E, F, Additional file 2: Fig. S1H). The protein and mRNA expression of GPX4 and haem oxygenase 1 (HO-1) were significantly changed after siRNA-P2RX7 transfection in the Erastin group (Fig. 4G–K). Ferroptosis is a ROS-dependent form of cell death, and lipid peroxidation is one of the main biochemical characteristics [53]. We found that inhibition of P2RX7 reduced MDA levels (Fig. 4L), ROS and cell death (Fig. 4O–R) but increased SOD and GSH levels (Fig. 4M, N). These results suggested that P2RX7 might mitigate cell death by regulating ferroptosis and oxidative stress.

### P2RX7 knockdown can reduce ferroptosis and oxidative stress in vivo

**Fig. 5:**
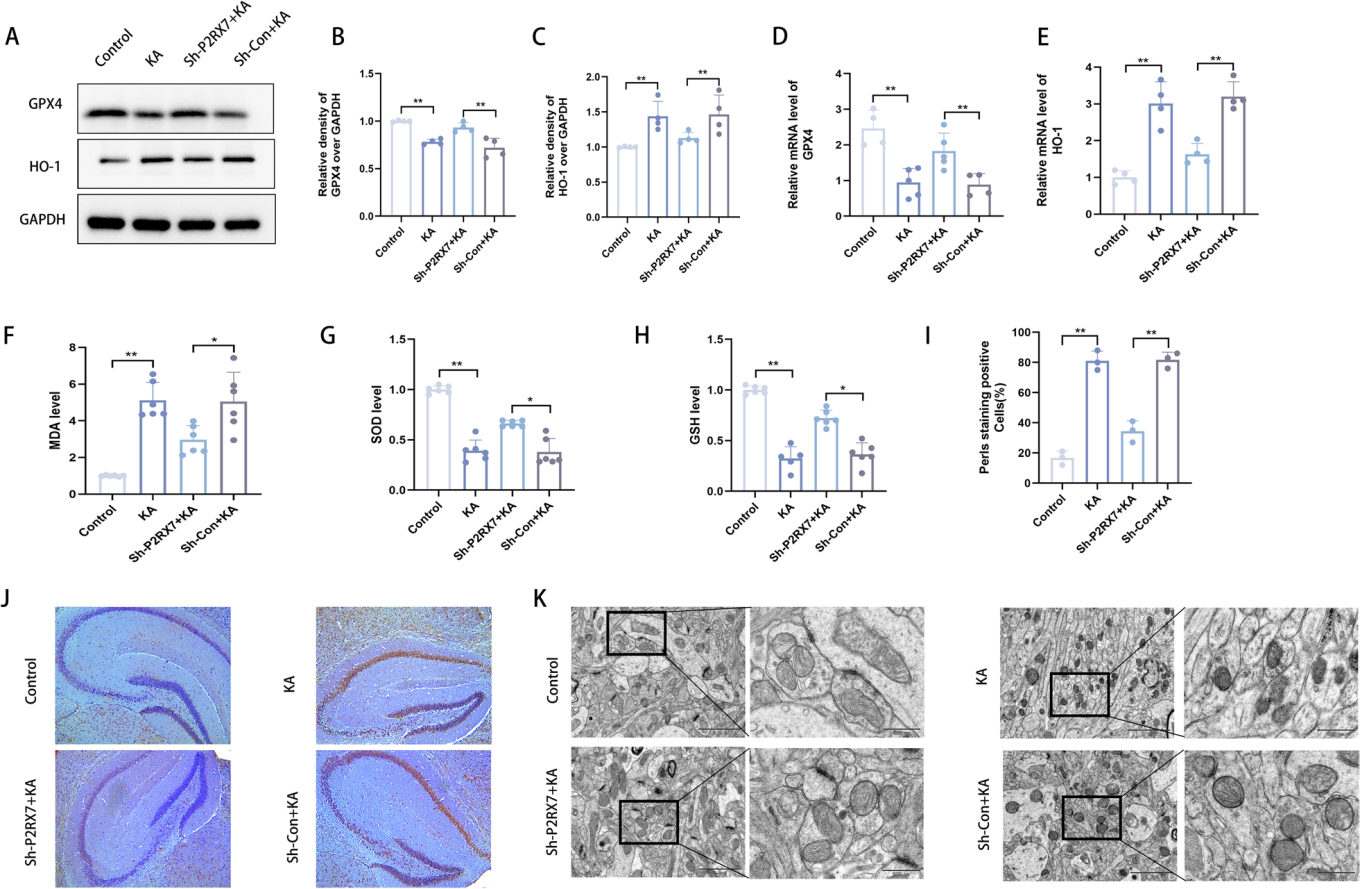
P2RX7 knockdown can reduce ferroptosis and oxidative stress in vivo*.* **A**–**C** Western blots and quantification of the protein levels of GPX4 and HO-1 in the control, KA, sh-P2RX7 + KA and sh-Con + KA groups. **D**, **E** Representative quantification of relative mRNA levels of GPX4 (**D**) and HO-1 (**E**) in the control, KA, sh-P2RX7 + KA and sh-Con + KA groups. **F**–**H** MDA, SOD and GSH assays to detect lipid peroxidation levels in hippocampal tissues from the control, KA, sh-P2RX7 + KA and sh-Con + KA groups. **I**, **J** Representative images of hippocampal areas by Perl’s staining in randomly selected hippocampal sections and semiquantitative analysis of Perl’s staining positive cells (%). Scale bar = 200 μm. **K** Representative TEM images of hippocampal areas for mitochondrial changes in randomly selected tissues. Scale bar = 200 μm or 100 μm. All data are expressed as the mean ± SD. *p < 0.05, **p < 0.01

To explore the reason for the increase in P2RX7 in epilepsy, transcriptional and translational levels were measured by qRT-PCR and Western blots, which showed that GPX4 was upregulated (Fig. 5A, B, D), whereas HO-1 was downregulated (Fig. 5A, C, E) after inhibiting P2RX7 in an epilepsy mouse model. A reduction in MDA level and increases in SOD and GSH levels were observed in response to P2RX7 inhibition compared sh-P2RX7 + KA to sh-Con + KA group (Fig. 5F–H). This is consistent with the results of the cell experiments. Iron accumulation is one of the main biochemical characteristics (two main biochemical characteristics: lipid peroxidation and iron accumulation) [53]. Prussian blue staining (Perl’s Iron Staining) is a common method used by pathologists to detect iron in biopsy specimens. Additionally, Perl’s Iron Staining results showed that the initially elevated level of iron accumulation was reduced in mice hippocampal tissue with P2RX7 inhibition (Fig. 5I, J). Mitochondria are the main energy supply organs of ATP. Sterol carrier protein 2-mediated trafficking of peroxidized lipids to mitochondria promotes GPX4 depletion-induced ferroptosis, supporting a role for mitochondria in ferroptosis [54]. Next, hippocampal tissues from mice were subjected to transmission electron microscopy to elucidate the mitochondrial membrane state after P2RX7 inhibition. Under transmission electron microscopy, we observed that KA-induced hippocampal mitochondria became smaller, the membrane density increased, and the cristae decreased. These are the typical electron microscopic characteristics of ferroptosis. Interestingly, inhibiting P2RX7 significantly improved the changes in mitochondrial morphology, leading to the majority of mitochondria remaining their normal form (Fig. 5K). Taken together, these results obtained from the epilepsy mouse model are consistent with those obtained in vitro, emphasizing that P2RX7 alleviates seizures by inhibiting neuronal ferroptosis.

### Inhibition of P2RX7 involves the MAPK signaling pathway

**Fig. 6:**
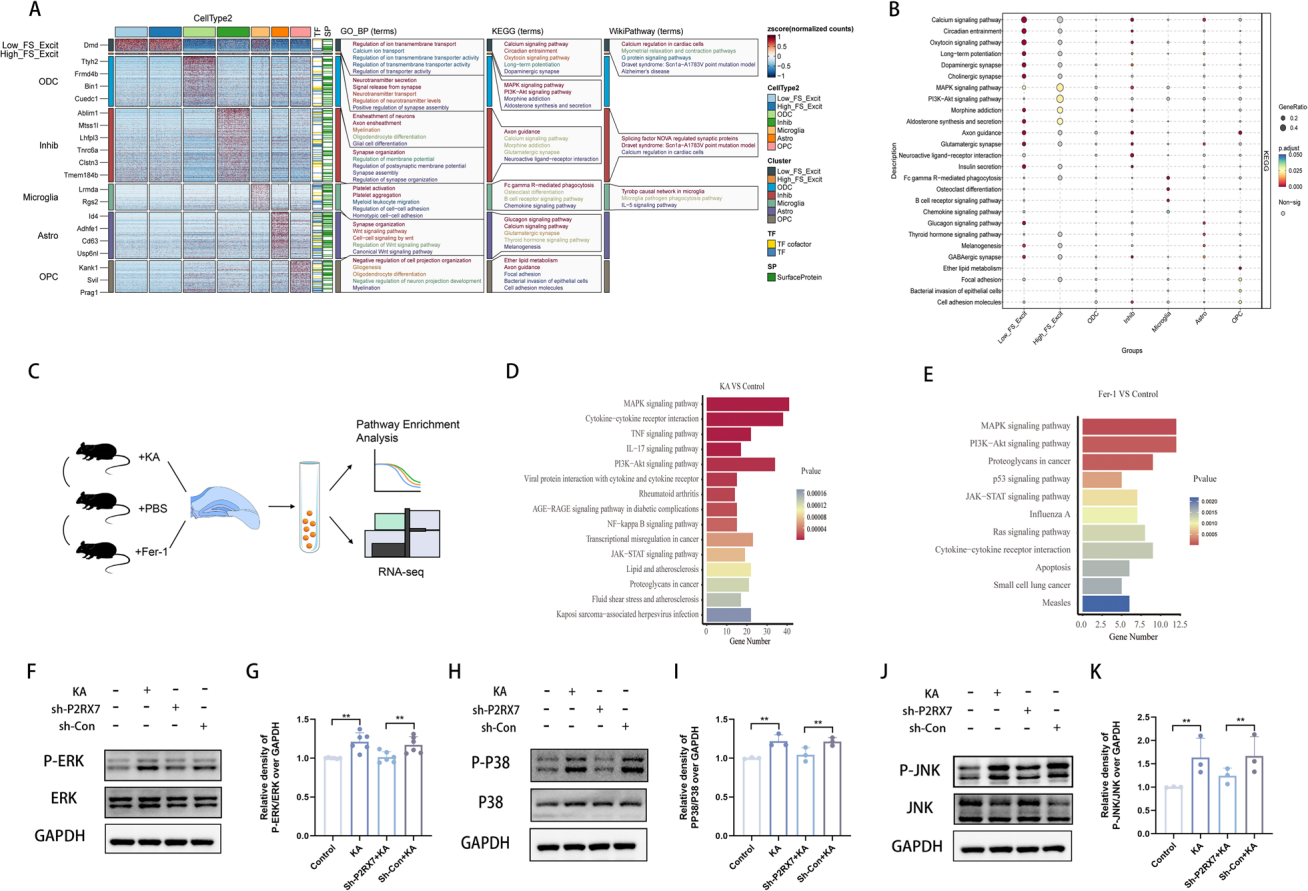
Inhibition of P2RX7 involves the MAPK Signaling Pathway. **A** Enrichment analysis was performed utilizing differentially expressed transcription factors and surface proteins derived from the DEGs extracted from seven distinct cellular subgroups (Excitatory neurons were split into Low-Ferro (Low-FS-Excit) and High-Ferro (High-FS-Excit) categories based on the Ferroptosis scores) in GSE143560. The bar chart shows the significantly enriched GO, KEGG, and WikiPathway terms. **B** Enrichment analysis was performed utilizing the DEGs extracted from seven distinct cellular subgroups. Notably, the MAPK signaling pathway emerged as the most significant pathway in the High-FS-Excit group (both in panels **A** and **B**). **C** A schematic diagram of transcriptome sequencing (these originate data from our murine model hippocampus tissues). **D**, **E** KEGG pathway enrichment analysis for DEGs in the KA (**D**) and Fer-1 (**E**) groups. The MAPK signaling pathway was the most significantly enriched in the KA (**D**) and Fer-1 (**E**) groups. **F**, **G** Western blots and quantification of the protein levels of P-ERK and ERK in the control, KA, sh-P2RX7 + KA and sh-Con + KA groups. **H**, **I** Western blots and quantification of the protein levels of P-P38 and P38 in the control, KA, sh-P2RX7 + KA and sh-Con + KA groups. **J**, **K** Western blots and quantification for comparing the protein levels of P-JNK and JNK in control, KA, sh-P2RX7 + KA and sh-Con + KA groups. The ratios of P-ERK/ERK, P-P38/P38 and P-JNK/JNK were increased in the KA group compared to control group, but decreased in the sh-P2RX7 + KA group compared to the sh-Con + KA group

To discover potential signaling pathways by which P2RX7 could regulate ferroptosis, the GSE143560 database was divided into 7 groups of transcription factor (TF) and surface protein (SP) based on the 7 major cell types (because excitatory neurons were divided into Low-Ferro score excit (Low-FS-Excit) and High-Ferro score excit (High-FS-Excit) groups). Further differential expression analysis and functional annotation of the enriched GO_BP terms, KEGG terms and WikiPathway terms indicated that the MAPK signaling pathway is one of the most likely pathways involved in the P2RX7 axis (Fig. 6A, Additional file 2: Fig. S1I). Subsequently, according to KEGG pathway analysis, the MAPK signaling pathway was the most significantly enriched pathway in the High-FS-Excit group (Fig. 6B). Previous experiments have shown the role of the MAPK signaling pathway in an epilepsy model [33], and the MAPK signaling pathway is closely connected to ferroptosis [55]. For systematic research, 5 mice were randomly selected for injection with PBS, 5 mice were injected with KA, and 5 mice were injected with Fer-1 (a ferroptosis inhibitor) in bilateral hippocampal tissues. The bilateral hippocampal tissues were immediately isolated, transcriptome sequencing was performed on hippocampal tissues (Fig. 6C), and KEGG pathway enrichment analysis was subsequently conducted (Fig. 6D, E). The genes upregulated in the KA group vs. the control group were mainly enriched in the MAPK signalling pathway (Fig. 6D). The gene upregulated in the Fer-1 group vs. the control group were mainly enriched in the MAPK and PI3K-Akt signalling pathways (Fig. 6E). Thus, the intersection of the transcriptome results indicated the importance of the MAPK signalling pathway. In general, the MAPK signalling pathway includes extracellular signal-related kinases (ERK1/2), jun amino-terminal kinases (JNK1/2/3), and p38-MAPK. This finding was verified at the protein level. First, compared to Control group, the KA group displayed significantly increased ratios of P-ERK/ERK, PP38/P38, and P-JNK/JNK (Fig. 6F–K). In contrast, compared to sh-Con + KA group, the sh-P2RX7 + KA group presented significantly reduced ratios (Fig. 6F–K). These results indicated that P2RX7 might influence epilepsy by regulating the MAPK pathway.

### P2RX7 facilitates ferroptosis resistance by regulating the MAPK/ERK signaling pathway

**Fig. 7:**
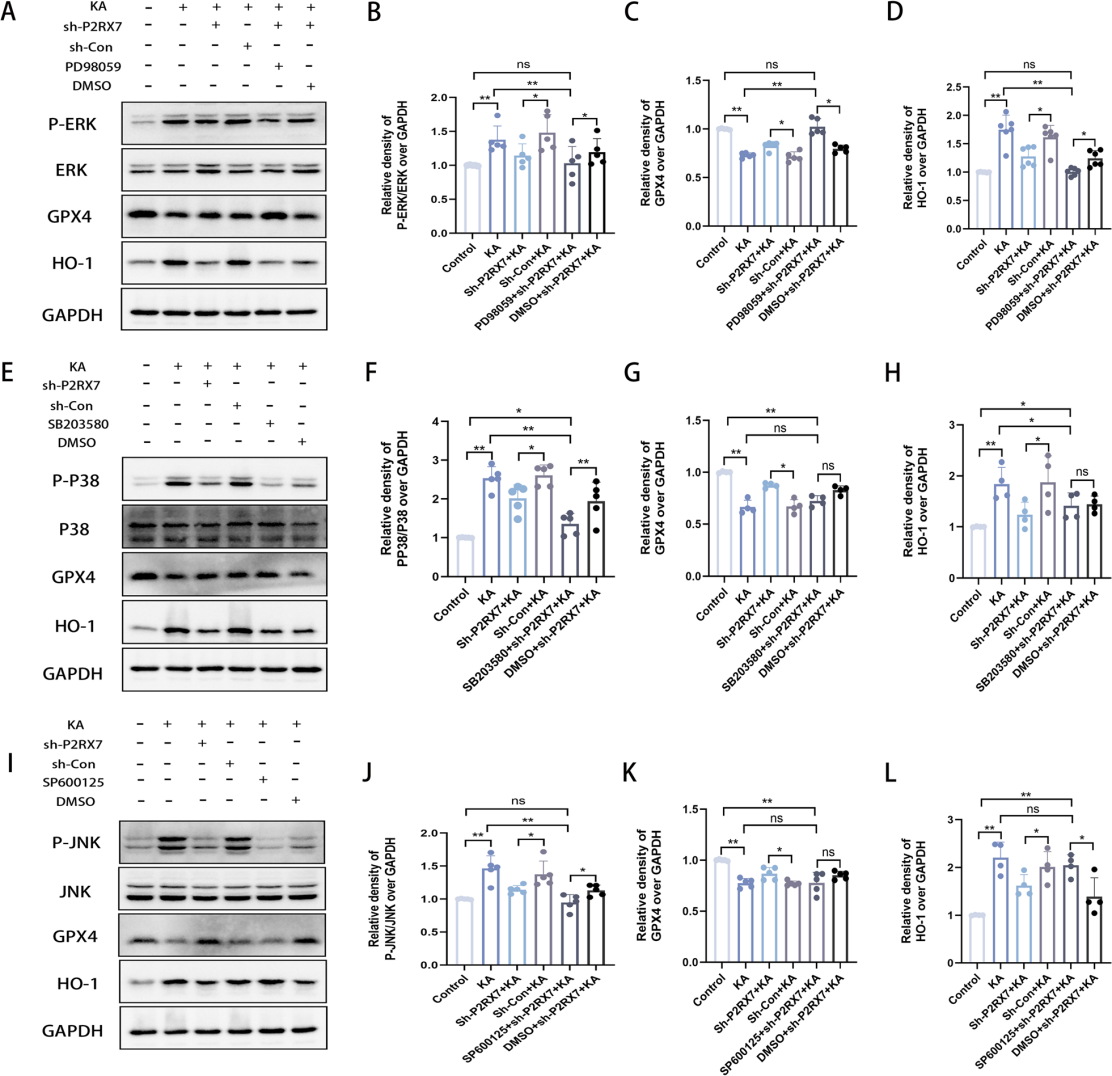
P2RX7 facilitates ferroptosis resistance by regulating the MAPK/ERK Signaling Pathway. **A**–**D** Western blots and quantification of the protein levels of P-ERK/ERK (**B**), GPX4 (**C**) and HO-1 (**D**) in the control, KA-induced epileptic, sh-P2RX7 + KA, sh-Con + KA, PD98059 + sh-P2RX7 + KA and DMSO + sh-Con + KA groups. **E**–**H** Western blots and quantification of the protein levels of P-P38/P38 (**F**), GPX4 (**G**) and HO-1 (**H**) in the control, KA-induced epileptic, sh-P2RX7 + KA, sh-Con + KA, SB203580 + sh-P2RX7 + KA and DMSO + sh-Con + KA groups. **I**–**L** Western blots and quantification for comparing the protein levels of P-JNK/JNK (**J**), GPX4 (**K**) and HO-1 (**L**) in control, KA-induced epileptic, sh-P2RX7 + KA, sh-Con + KA, SP600125 + sh-P2RX7 + KA and DMSO + sh-Con + KA groups. Representative quantification of relative protein levels of GPX4 and HO-1 were significantly changed after administration of ERK inhibitor (PD98059) compared to the KA group (**A**). There was no significant change in GPX4 and HO-1 after administration of a P38 inhibitor (SB203580) (**E**) or JNK inhibitor (SP600125) (**I**)

The regulation of the MAPK signaling pathway by P2RX7 in epilepsy was confirmed in our experiments (Fig. 6F–K). However, it is unclear through which cascade of the MAPK pathway does P2RX7 regulate ferroptosis? To address this question, the ERK inhibitor PD98059, the P38 inhibitor SB203580, the JNK inhibitor SP600125 and DMSO were injected into mice. In addition, P2RX7-AAV injection and subsequent KA injection were performed on mice. Following hippocampal injection, three or four mice injected with KA failed to survive, presumably because of the repetitive operation. We ensured that there were no less than 6 mice per group for subsequent western blot experiments. Notably, significant changes in the ferroptosis core proteins GPX4 and HO-1 were observed after ERK inhibitor administration, compared to the KA group (Fig. 7A–D). However, there was no significant difference in GPX4 and HO-1 after the administration of P38 (Fig. 7E–H) and JNK inhibitors (Fig. 7I–L). Thus, blocking P2RX7 might regulate GPX4/HO-1 axis by suppressing the MAPK-ERK pathway, participating in ferroptosis in epilepsy.

### Activation of miR-211-5p ameliorated ferroptosis and oxidative stress in hippocampal neurons in mice with in KA-induced epilepsy

**Fig. 8:**
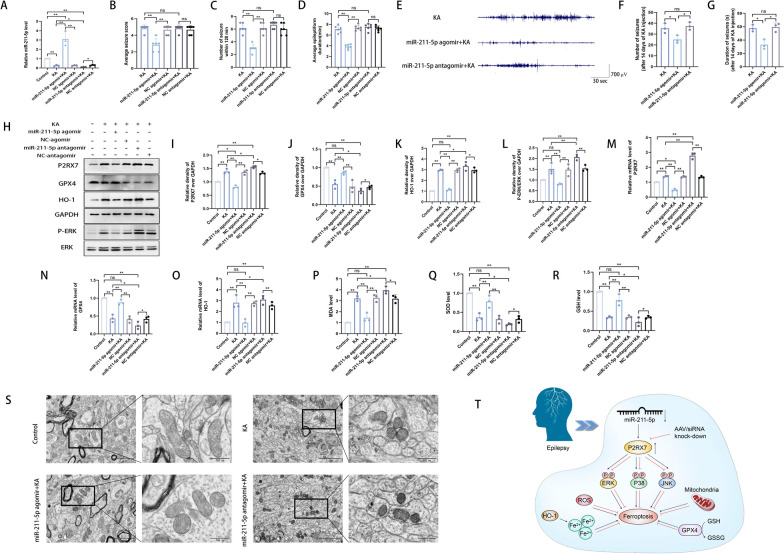
Activation of miR-211-5p ameliorated ferroptosis and oxidative stress in hippocampal neurons of mice with KA-induced epilepsy. **A** Relative miR-211-5p expression in the KA, miR-211-5p agomir + KA, NC agomir + KA, miR-211-5p antagomir + KA and NC antagomir + KA groups compared with the control group. **B**–**D** The average seizure-stage score was 4.83 ± 0.41 in the KA group vs. 2.83 ± 0.75 in the miR-211-5p agomir + KA group vs. 4.67 ± 0.52 in the NC agomir + KA group vs. 4.83 ± 0.41 in the miR-211-5p antagomir + KA group vs. 4.67 ± 0.52 in the NC antagomir + KA group (**B**). The number of seizures within 120 min (**C**, average of 6 in the non-miR-211-5p agomir + KA group vs. 3 in the miR-211-5p agomir + KA group) and the average epileptiform duration of the first seizure (**D**, 7.08 ± 0.67 in the KA group vs. 3.87 ± 0.42 in the miR-211-5p agomir + KA group vs. 7.35 ± 0.51 in the NC agomir + KA group vs. 7.50 ± 0.61 in the miR-211-5p antagomir + KA group vs. 7.23 ± 0.49 in the NC antagomir + KA group). **E** Representative 5-min EEG recordings from the hippocampus (after 14 days of KA injection) showed spontaneous electrographic seizures in the KA, the miR-211-5p agomir + KA and the miR-211-5p antagomir + KA group. **F** The number of SRS recorded after 14 days of KA injection, demonstrating that the miR-211-5p agomir + KA group had a lesser number of seizures than those of the KA and the miR-211-5p antagomir + KA group. **G** The mean SRSs duration of the miR-211-5p agomir + KA group was significantly shorter than that of the KA and the miR-211-5p antagomir + KA group. **H**–**L** Western blot analysis and quantification of the protein levels of P2RX7 (**I**), GPX4 (**J**), HO-1 (**K**) and P-ERK/ERK (**L**) in the control, KA-induced epilepsy, miR-211-5p agomir + KA, NC agomir + KA, miR-211-5p antagomir + KA and NC antagomir + KA groups. **M**–**O** Representative quantification of the relative mRNA levels of P2RX7 (**M**), GPX4 (**N**) and HO-1 (**O**) in the control, KA, miR-211-5p agomir + KA, NC agomir + KA, miR-211-5p antagomir + KA and NC antagomir + KA groups. **P**–**R** Measurement of MDA, SOD and GSH levels to evaluate lipid peroxidation in hippocampal tissues from the abovementioned six groups. **S** Representative TEM images of hippocampal areas for evaluation of mitochondrial changes in randomly selected tissues. Scale bar = 200 μm or 100 μm. **T** Hypothesized mechanisms by which the miR-211-5p/P2RX7/ERK/GPX4 axis regulates epilepsy-associated neuronal ferroptosis and oxidative stress. All data are expressed as the mean ± SD. *p < 0.05, **p < 0.01

The direct interaction between miR-211-5p and P2RX7 was verified as described above. To further explore the mechanism by which miR-211-5p regulates ferroptosis in mice with KA-induced epilepsy, a miR-211-5p agomir and a miR-211-5p antagomir were injected into hippocampal tissue. qRT-PCR was performed to detect differences in the level of miR-211-5p to assess the induction and inhibition efficiencies of miR-211-5p (Fig. 8A). To evaluate the anticonvulsant effect of miR-211-5p induction and inhibition, the seizure severity score, number of seizures within 120 min, and epileptiform duration of the first seizure were recorded. The mean seizure severity score, number and duration were not significantly different between among the KA group, the NC agomir + KA group and the NC antagomir + KA group, indicating that the viral vectors exerted no effect in the KA-induced epilepsy model. Compared with the KA group, the miR-211-5p agomir + KA group showed significant decreases in the average seizure score (2.83 ± 0.75 vs. 4.83 ± 0.41) (Fig. 8B), with an average of 3 seizures within 120 min (Fig. 8C) and the average epileptiform duration of the first seizure (3.87 ± 0.42 vs. 7.08 ± 0.67) (Fig. 8D). Compared with the KA group, the miR-211-5p antagomir + KA group showed a significant increase in the average epileptiform duration of the first seizure (7.50 ± 0.61 vs. 7.08 ± 0.67) (Fig. 8D). Moreover, the severity of epilepsy in the miR-211-5p antagomir + KA group reached stage 5, and the average of 6 seizures in this group was consistent with that in the KA group (Fig. 8B, C). The EEG results showed the mean number of SRSs in the miR-211-5p agomir + KA group was reduced compared to the KA group and miR-211-5p antagomir + KA group (Fig. 8E, F). The mean seizure duration of the miR-211-5p agomir + KA group was significantly shorter than that of the KA group and miR-211-5p antagomir + KA group (Fig. 3G). No significant differences were observed between the and miR-211-5p antagomir + KA group. Moreover, we further confirmed the mutual relationship between miR-211-5p and P2RX7 through WB and qRT-PCR analyses (Fig. 8H, I, M). To determine the mechanism of miR-211-5p, we showed through WB or qRT-PCR that GPX4 was upregulated (Fig. 8H, J, N), but HO-1 and P-ERK/ERK were downregulated (Fig. 8H, K, L, O) after induction of miR-211-5p in the epilepsy mouse model. A reduction in the MDA level and increases in the SOD and GSH levels were observed in response to miR-211-5p induction (Fig. 8P–R). By transmission electron microscopy, we observed that induction of miR-211-5p significantly attenuated the changes in mitochondrial morphology, with most mitochondria retaining a normal morphology (Fig. 8S). After inhibiting miR-211-5p, effects opposite those of miR-211-5p induction were observed in the epilepsy mouse model. Thus, we hypothesize that the microRNA-211-5p/P2RX7/ERK/GPX4 axis might regulate epilepsy-associated neuronal ferroptosis and oxidative stress (Fig. 8T).

## Discussion

The aetiology of epilepsy is very complex, and the pathogenesis of epilepsy remains unclear. Accumulating evidence suggests that iron is involved in epilepsy. For example, iron metabolism is related to seizures in adults with epilepsy [56]. Additionally, dysregulation of iron accumulation and iron metabolism are associated with epilepsy and seizures after temporal lobe epilepsy [57]. These studies underscore the importance of iron in epilepsy. However, ferroptosis, a new type of programmed cell death, is attributed to an imbalance in oxidative stress caused by iron ion accumulation. Targeting ferroptosis has been proposed as a promising therapeutic strategy for epilepsy [58]. In addition, vitamin E [59] and lapatinib [60] might alleviate epilepsy by impeding ferroptosis. Nonetheless, no studies have proven how ferroptosis contributes to epilepsy and what targets can effectively regulate ferroptosis in epilepsy.

MicroRNAs are endogenous noncoding ribonucleotides. Over the past 20 years, miRs have fundamentally changed scientists’ understanding of gene regulatory networks, which is a new interdisciplinary direction in neuroscience research [61]. The multitargeted ability of miRs provides an advantage for exploring epilepsy with complex pathophysiology. Therefore, differential expression of miRs may participate in neurological diseases [62]. In this study, we screened epilepsy-related miRs, and identified downregulation of miR-211-5p in epileptic patients. Abnormal downregulation of miR in hippocampal tissues of epileptic mice and blood samples of epileptic patients could be verified by qRT-PCR. MiR-211-5p has been indicated in the physiology and pathology of epilepsy [11]. Therefore, how does miR-211-5p affect the development of epilepsy? Generally, miR-211-5p could bind to several genes, including CACNA1C, P2RX7, TMTC2, JPH3 and GRM1. MiRs bind to complementary mRNAs and prevent their translation, resulting in a decrease in their protein levels. Among these genes, CACNA1C, JPH3 and GRM1 showed the same trend as miR-211-5p. In contrast, P2RX7 and TMTC2 presented a trend opposite that of miR-211-5p, so we only focused on these upregulated genes in epilepsy. While it is possible that miR-211-5p may also affect epilepsy by targeting TMTC2, our current study primarily assessed P2RX7, which displayed the most significant upregulation as measured by qRT-PCR. Therefore, we hypothesized that the upregulation of P2RX7 in epilepsy was attributed to the downregulation of miR-211-5p.

P2RX7 is a nonselective ligand-gated homotrimeric cation channel activated by extracellular adenosine triphosphate (ATP), which has been proposed as a possible drug target [25]. In addition, P2RX7 receptor signaling components may serve as biomarkers for the diagnosis of TLE [29]. According to data analysis and validation in the blood of patients and in vivo experiments, our study revealed that the expression of P2RX7 is upregulated. A stereotactic apparatus was applied to inject AAV into the hippocampus to knock down P2RX7 in animal models. Interestingly, P2RX7 inhibition alleviated seizures. However, the exact reasons for the upregulation of P2RX7 expression in epilepsy are not fully understood, but several hypotheses and mechanisms can explain this phenomenon, such as high ATP release [63], increased intracellular calcium levels [64], elevated levels of inflammation [47] and regulation by microRNAs [65].

Recent studies have shown a strong correlation between P2RX7 and ferroptosis in acute ischemic stroke [66] and comorbid chronic pain and depression behaviour [67], but according to data analysis and validation in vitro and in vivo experiments, our study was the first to find the relationship between P2RX7 and ferroptosis in epilepsy. Indeed, activation of P2RX7 leads to the activation and release of inflammatory factors from microglia [68], as well as the modulation of neurotransmitter release from neurons [69]. However, in our study, P2RX7 was predominantly expressed in neurons, followed by microglia, with excitatory neurons exhibiting greater ferroptosis activity than other subsets. Thus, P2RX7 alleviates seizures by inhibiting excitatory neuronal ferroptosis.

To illustrate potential signaling pathways through which P2RX7 mediates ferroptosis and alleviates epilepsy, we conducted transcriptome sequencing analysis and identified significant changes in the mitogen-activated protein kinase (MAPK) cascade. The MAPK signaling pathway plays a crucial role in various physiological and pathological processes [70], including proliferation, migration, apoptosis, inflammation and oxidative stress [71, 72]. The generic MAPK signaling pathway is shared by four distinct cascades, including ERK1/2, JNK1/2/3, p38-MAPK and ERK5. Highly conserved kinase cascades linking transmembrane receptors to downstream effectors are activated in response to various physiological or pathological stimuli associated with synaptic activity, plasticity and neuronal activation [73–75]. For instance, CB2R could be induced by ERK and P38 to protect against the onset of epilepsy [76]. In addition, Small et al. [77] and Myers et al. [78] demonstrated that JNK signaling might be involved in the pathogenesis of epilepsy. Collectively, the MAPK pathway is critically involved in epilepsy. However, whether P2RX7 regulates ferroptosis by regulating the MAPK pathway remains unknown. In our study, the MAPK pathway was activated in epileptic mice, which was reversed by inhibiting P2RX7. To investigate which cascades are more effective in regulating ferroptosis under AAV-P2RX7 conditions, specific inhibitors of ERK (PD98059), P38 (SB203580) and JNK (SP600125) were employed. As previously described, activation of P2RX7 might mediate the production of NOX2-dependent ROS by activating extracellular ERK [79]. Consistently, blocking P2RX7 attenuated ferroptosis in the endothelium and reduced HG-induced haemorrhagic transformation after MCAO by inhibiting ERK1/2 [80]. In our study, P2RX7 blockade regulated GPX4/HO-1 by suppressing the ERK cascade in murine models of epilepsy.

In terms of neurological diseases, downregulation of miR-211-5p was found in rat models of depression [81] and cerebral ischemia reperfusion injury [82]. A previous study found that the neurotherapeutic effect of empagliflozin downregulates miR-211-5p, resolving oxidative stress by suppressing the PERK/CHOP ER stress pathway in a PD model [83]. Currently, only one study has demonstrated that dynamic decreases miR-211-5p expression induce hypersynchronization and both nonconvulsive and convulsive seizures [11]. Our study is the first to find the relationship between miR-211-5p and ferroptosis in mice with KA-induced epilepsy. The inhibitory effects of miR-211-5p on seizures are likely associated with its inhibitory effect on ferroptosis, as (1) activation of miR-211-5p or knockdown of P2RX7 increases the GPX4 expression level and the SOD and GSH concentrations but decreases the HO-1 expression level and MDA concentration and mitochondrial membrane density, and (2) activation of miR-211-5p or knockdown of P2RX7, both of which suppress the ERK cascade in murine models of epilepsy and are closely associated with the pathogenesis of ferroptosis and oxidative stress, also alleviates seizures. These are very important findings, although other target genes of miR-211-5p or other enriched pathways need to be carefully evaluated in epilepsy models. In addition, we acknowledge the important of considering the potential impact of the vehicle (DMSO) in future studies, and we recognize that comparing Fer-1 with a solvent control can provide additional insights into the observed effects.

## Conclusion

In summary, a direct interaction exists between miR-211-5p and P2RX7. Accordingly, P2RX7 blockade can regulate GPX4/HO-1 by suppressing the MAPK/ERK pathway. Consequently, this network modifies ferroptosis in epilepsy.

### Supplementary Information

**Additional file 1: Table S1.** The clinical data of the epilepsy patients. **Table S2.** Primer sequence.

**Additional file 2: Figure S1.** **A** The transcriptome data of GSE99455 abstracted from hippocampal miRNA profiling of intractable epilepsy and healthy controls. MiR-211-5p was downregulated in adult-onset epilepsy patients vs. controls. **B** MiR-211-5p was downregulated in adolescence-onset epilepsy patients vs. controls. **C** the RNA sequence alignment showed that the 3′-UTR of P2RX7 mRNA contained a complementary site for the seed region of miRNA-211-5p. **D** The relative mRNA levels of miRNA-211-5p in mimic and mimic nc groups for 50 nm/24 h, 100 nm/24 h, 50 nm/48 h, 100 nm/48 h were measured by qRT-PCR. **E** The relative mRNA levels of miRNA-211-5p in inhibitor and inhibitor nc groups for 50 nm/24 h, 100 nm/24 h, 50 nm/48 h, 100 nm/48 h were measured by qRT-PCR. **F**, **G** Western blots and quantification of the protein levels of P2RX7 in the HT22 and SH-SY5Y cells. **H** The relative mRNA levels of P2RX7 in siRNA-P2RX7 and siRNA-NC groups for 50 nm/24 h, 100 nm/24 h, 50 nm/48 h, 100 nm/48 h were measured by qRT-PCR. **I** Enrichment analysis was performed utilizing the DEGs extracted from seven distinct cellular subgroups (GO terms). All data are expressed as the mean ± SD. *p < 0.05, **p < 0.01.

## Data Availability

Data supporting the present study are available from the corresponding author upon reasonable request.

## Abbreviations

GPX4: Glutathione peroxidase 4
P2RX7: Purinergic receptor P2X
ATP: Extracellular adenosine triphosphate
TLE: Temporal lobe epilepsy
AAV: Adeno-associated virus
KA: Kainic acid
GEO: Gene Expression Omnibus
DEG: Differential gene expression
tSNE: T-distributed Random Neighbour Embedding
HO-1: Haem oxygenase 1
TF: Transcription factor
SP: Surface protein
MAPK: Mitogen-activated protein kinase
