# Supplementary material for: The microRNA-211-5p/P2RX7/ERK/GPX4 axis regulates epilepsy-associated neuronal ferroptosis and oxidative stress
Source: J Neuroinflammation. 2024 Jan 8;21:13. doi: 10.1186/s12974-023-03009-z (PMC10773122; doi:10.1186/s12974-023-03009-z)
Supplement: Supplementary file 1 — Additional file 1: Table S1. The clinical data of the epilepsy patients. Table S2. Primer sequence. [file 12974_2023_3009_MOESM1_ESM.docx]

**Table S1. Characteristics of patients with epilepsy overall**

| Sample characteristics | Total sample(N=60) |
| --- | --- |
| Female sex (%) | 29 (48.3) |
| Mean age (years) (SD)  Duration of epilepsy (years) (SD) | 33.53(9.49)  12.35(9.56) |
| Age at onset of epilepsy (years) (SD) | 21.18(10.21) |
| Type of seizure  Focal Onset (%)  Generalized Onset (%)  Etiology(structural/metabolic)  Others  EEG (within 3 month)  Abnormal but without epileptiform abnormalities  Abnormal with epileptiform abnormalities  MRI（within 6 month）  Normal  Abnormal  No. of AEDs  2  ＞＝3 | 17(28.3)  43(71.7)  22(36.7)  38(63.3)  23(38.3)  37(61.7)  29(48.3)  31(51.6)  54(90.0)  6(10.0) |

Values are expressed as a number (%) ; AEDs, antiepileptic drugs

**Table S2. Primer sequence**

Human

H-GPX4-F: CCCGATACGCTGAGTGTGGTTTG

H-GPX4-R: CCTTGCCCTTGGGTTGGATCTTC

H-HMOX-1-F: TGCCAGTGCCACCAAGTTCAAG

H-HMOX-1-R: TGAGTGTAAGGACCCATCGGAGAAG

H-P2X7-F: AGAGGAGATCGTGGAGAATGGAGTG

H-P2X7-R: GCACCAGGCAGAGACTTCACAG

H-TMTC2-F: CCGCACTCTCACCTTCTTCTACTTG

H-TMTC2-R: TCTGTCTACGCTCGGGCTCTTC

H-GAPDH-F: CAAGGCTGTGGGCAAGGTCATC

H-GAPDH-R: GTGTCGCTGTTGAAGTCAGAGGAG

Mouse

M-GPX4-F: CCCGATATGCTGAGTGTGGTTTACG

M-GPX4-R: TAGAGATAGCACGGCAGGTCCTTC

M-HMOX-1-F: ACACAGCACTATGTAAAGCGTCTCC

M-HMOX-1-R: GTTGAGCAGGAAGGCGGTCTTAG

M-P2X7-F: CCAAGGTCAAAGGCATAGCAGAGG

M-P2X7-R: TAGGACACCAGGCAGAGACTTCAC

M-TMTC2-F: CCCTGAGTGCTGATTTCTGCTACG

M-TMTC2-R: GCCTTGGAGAAGCGTGTGAAGAG

M-GAPDH-F: TCACCATCTTCCAGGAGCGAGAC

M-GAPDH-R: TGAGCCCTTCCACAATGCCAAAG
