# Supplementary figures and images for: The microRNA-211-5p/P2RX7/ERK/GPX4 axis regulates epilepsy-associated neuronal ferroptosis and oxidative stress
Source: J Neuroinflammation. 2024 Jan 8;21:13. doi: 10.1186/s12974-023-03009-z (PMC10773122; doi:10.1186/s12974-023-03009-z)

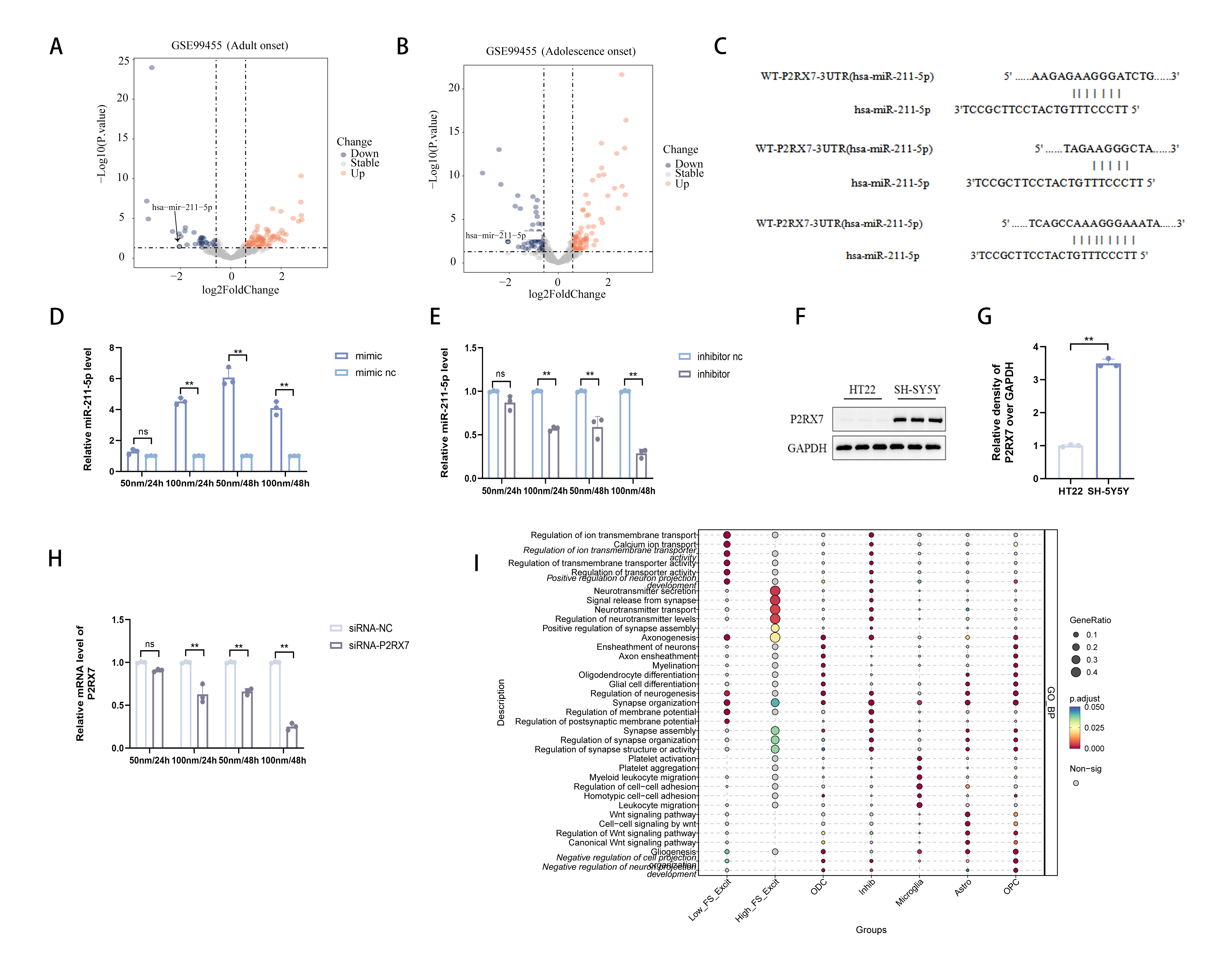

Supplement: Supplementary file 2 — Additional file 2: Figure S1. A The transcriptome data of GSE99455 abstracted from hippocampal miRNA profiling of intractable epilepsy and healthy controls. MiR-211-5p was downregulated in adult-onset epilepsy patients vs. controls. B MiR-211-5p was downregulated in adolescence-onset epilepsy patients vs. controls. C the RNA sequence alignment showed that the 3′-UTR of P2RX7 mRNA contained a complementary site for the seed region of miRNA-211-5p. D The relative mRNA levels of miRNA-211-5p in mimic and mimic nc groups for 50 nm/24 h, 100 nm/24 h, 50 nm/48 h, 100 nm/48 h were measured by qRT-PCR. E The relative mRNA levels of miRNA-211-5p in inhibitor and inhibitor nc groups for 50 nm/24 h, 100 nm/24 h, 50 nm/48 h, 100 nm/48 h were measured by qRT-PCR. F, G Western blots and quantification of the protein levels of P2RX7 in the HT22 and SH-SY5Y cells. H The relative mRNA levels of P2RX7 in siRNA-P2RX7 and siRNA-NC groups for 50 nm/24 h, 100 nm/24 h, 50 nm/48 h, 100 nm/48 h were measured by qRT-PCR. I Enrichment analysis was performed utilizing the DEGs extracted from seven distinct cellular subgroups (GO terms). All data are expressed as the mean ± SD. *p < 0.05, **p < 0.01. [file 12974_2023_3009_MOESM2_ESM.tif]
